# Supplementary material for: Hyperspectral imaging in animal coloration research: A user-friendly pipeline for image generation, analysis, and integration with 3D modeling
Source: PLoS Biol. 2024 Dec 3;22(12):e3002867. doi: 10.1371/journal.pbio.3002867 (PMC11614258; doi:10.1371/journal.pbio.3002867)
Supplement: S1 Appendix — Raw data and code for all supplementary figures are available (see the Data availability section) (.PDF). (PDF) [file pbio.3002867.s001.pdf]

Supporting Information for Hyperspectral imaging in animal coloration research: a user-friendly pipeline for image generation, analysis and integration with 3D modeling

Benedict G. Hogan, Mary Caswell Stoddard

Contents

Specimens 3

Table A. Details of the specimens used in the analysis. Specimens were loaned by the American Museum of Natural History (New York City, USA). 3

Light set-up diagram 4

Figure A. Diagram indicating the physical arrangement of the imager, specimen, and light source during data collection 4

Spectral filtering and extrapolation 5

Figure B. Comparison of unfiltered and filtered sampled spectra. 5

Extrapolation on natural spectra 6

Figure C. Extrapolation between 300 and 325nm applied to full (300-700nm) spectrophotometric data. 7

Consequences of low spectral resolution 8

Figure D. Consequences of low spectral resolution in natural reflectance spectra 9

Embedding sampled spectra into a KLPD visual system-independent color space 10

Figure E. Version of main text Fig 5, here UMAP embedding is applied to the combined brightness (dW) and shape (dSh) components of KLPD 11

Figure F. Version of main text Fig 5, here UMAP embedding is applied to only the shape (dSh) component of KLPD 11

UMAP parameterization 12

Figure G. Visual system-independent embedding of sampled spectra for each patch, using alternative settings for UMAP embedding (minimum distance = 1, versus 0.1 in the main text) 12

Figure H. Visual system-independent embedding of sampled spectra for each patch, using alternative settings for UMAP embedding (number of neighbors= 2000, versus 15 in the main text) 13

Embedding sampled spectra into avian & human visual models 14

Generating and comparing chromatic and achromatic color contrasts 15

Figure I. Plot of the chromatic (dS) and achromatic (dL) contrasts between the median spectrum from each patch of the specimens 15

Phylogeny 16

Table B. Table of GenBank accession codes for genes for each specimen and species found in the subfamily Paradisaeinae 16

Figure J. Bayesian maximum likelihood consensus tree from which main text Fig 2 was generated, showing all specimens for each species and with all genera expanded. 18

Sampling and patches 19

Figure K. Images indicating the location of samples for each of the 'Whole' body plans in the main paper 19

Figure L. Images indicating the location of samples for each of the ‘Back’ patches in the main paper 20
Figure M. Images indicating the location of samples for each of the ‘Shoulder’ patches in the main paper 21
Figure N. Images indicating the location of samples for each of the ‘Belly’ patches in the main paper 22
Figure O. Images indicating the location of samples for each of the ‘Breast’ patches in the main paper 23
Additional plots 24
Figure P. Version of main text Fig 6, where the z-axis of the tetrahedral avian color space has not been collapsed to 2D 24
Figure Q. Visual system-dependent embedding of sampled spectra for each patch, using the receptor noise limited (RNL) color space (as defined in [13]), using visual sensitivities, cone densities and Weber fractions as described in the ‘Generating and comparing chromatic and achromatic color contrasts’ section) 25
3D Model 26
Figure R. Diagram of the set-up used during photogrammetry, showing the specimen resting on a custom designed stand with calibration targets, mounted onto a rotating stage 26
Figure S. Screen capture from Metashape (v2.0.2, Agisoft LLC, Saint Petersburg, Russia) 26
Figure T. Renders of the 3D models for each specimen, showing the dorsal side 27
Tail morphology model 28
Figure U. Analyzing the shapes of bird-of-paradise tails 28
Fluorescence 29
Figure V. On the advice of Glenn Seeholzer, we photographed specimens under a small blacklight torch, (390nm - 395nm emission). The Magnificent bird-of-paradise’s shoulder/mantle patch fluoresces a bright off-white under this lighting 29
References 30

Specimens

Table A. Details of the specimens used in the analysis. Specimens were loaned by the American Museum of Natural History (New York City, USA).

Catalogue Number	Species	Date collected
SKIN 678668	*Cicinnurus regius*	Unknown
SKIN 303134	*Cicinnurus regius*	3/22/1931
SKIN 294486	*Cicinnurus magnificus*	4/27/1928
SKIN 678383	*Cicinnurus magnificus*	7/12/1928
SKIN 678503	*Cicinnurus magnificus x Cicinnurus regius*	Unknown
SKIN 294492	*Cicinnurus magnificus x Cicinnurus regius*	7/??/1928

Light set-up diagram

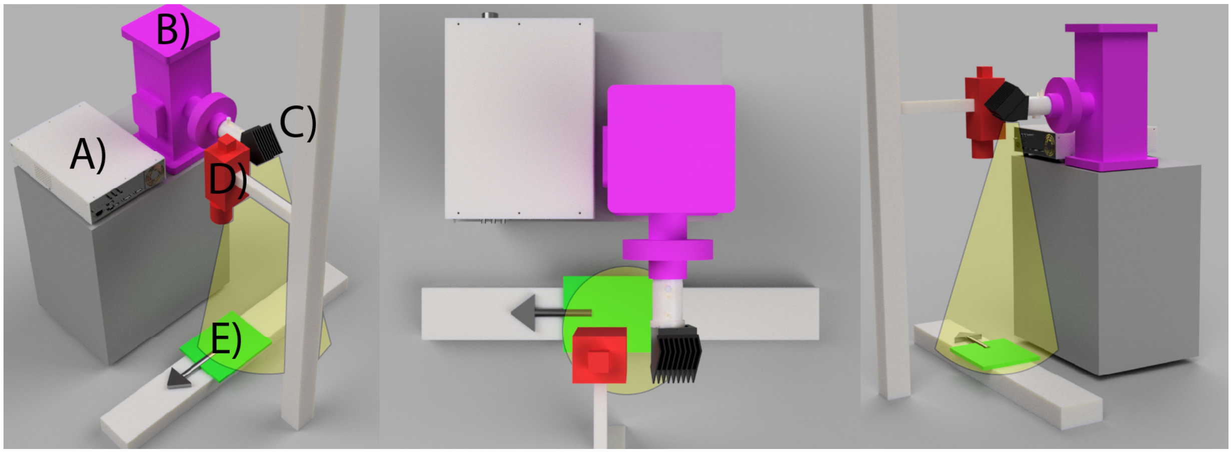

Figure A. Diagram indicating the physical arrangement of the imager, specimen, and light source during data collection. A) Power was supplied and an electronic shutter controlled by means of a unit mounted next to the light source housing. B) Broad-band light was produced by a 450W Xenon ozone free arc lamp in a housing (purple) with a horizontal light port. C) The light (yellow) was passed through a liquid filter and a turning mirror (black) to bend the light toward the sample. D) The hyperspectral imager (red) and turning mirror (black) were placed as close to one another as possible to minimize the angle between incident light and light reflected into the imager. The specimen was placed on the linear translation stage (green) and slowly moved across the (line-shaped) field of view of the imager while exposures were collected. At all times, the user ensured that while the light was on, a thick curtain was closed between the user and light source to minimize UV exposure of the user.

Spectral filtering and extrapolation

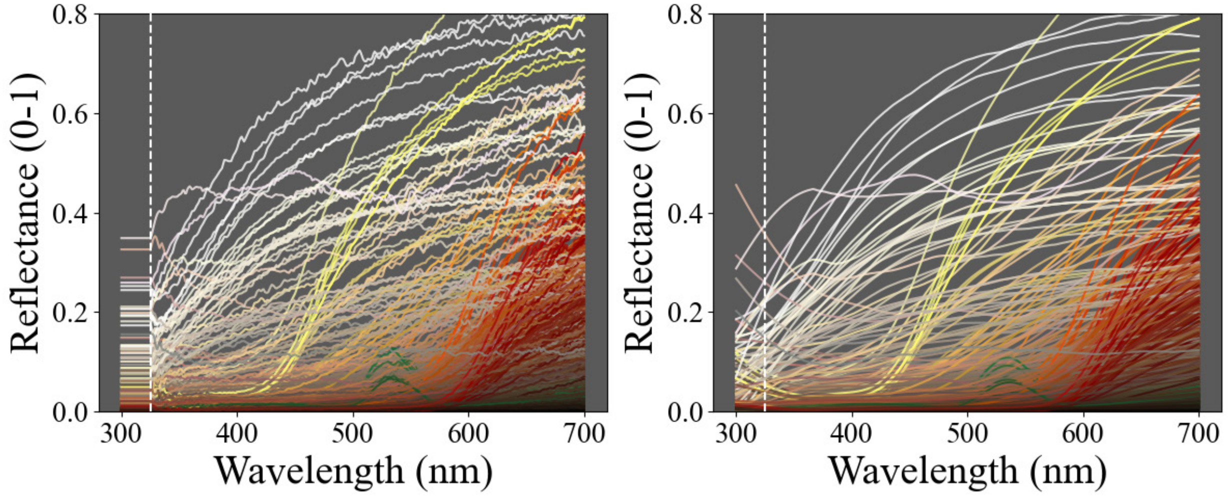

Figure B. Comparison of unfiltered and filtered sampled spectra. In the left panel, sampled spectra (collected as the median of a 10x10 pixel region of hyperspectral image) from the 'whole' patch across all specimens without filtering or extrapolation. The right-hand panel shows the same data but filtered using a Savitzky-Golay filter (window 45nm, polynomial order 2), with extrapolation between 300 and 325nm using a cubic 1D monotonic smoothing spline fit to the data from 325-700nm for each spectrum. The spectra are colored by approximate appearance to humans. The data underlying this figure can be found at https://doi.org/10.5061/dryad.j0zpc86nf.

Extrapolation on natural spectra

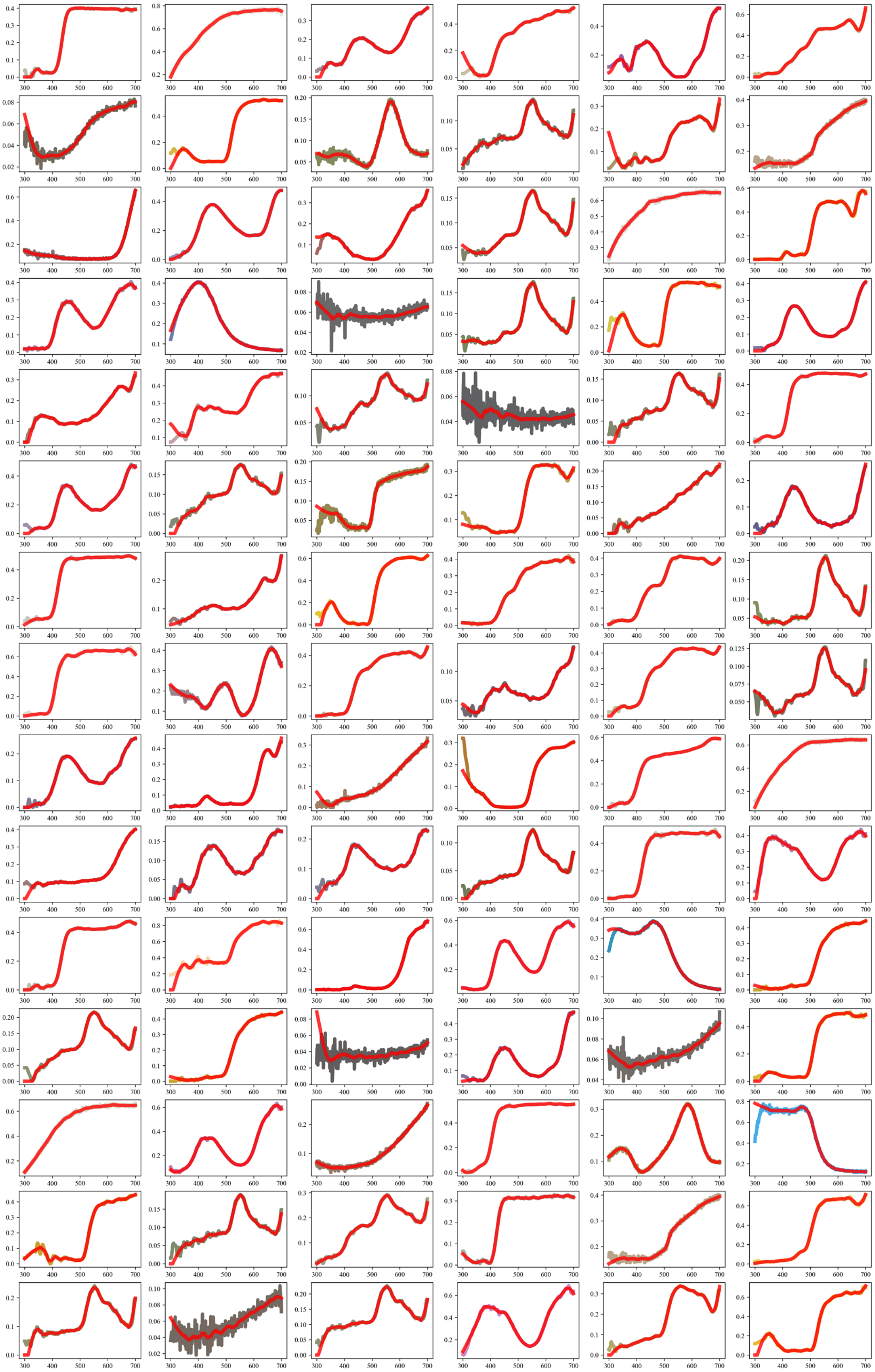

Figure C. Extrapolation between 300 and 325nm applied to full (300-700nm) spectrophotometric data. To demonstrate the effects of extrapolation and filtering, we overlay a set of natural reflectance spectra (colored lines) with a version of each spectrum that has been filtered and extrapolated between 300 and 325 nm (as in the main text). Because the original data contains measured values for this range, this plot allows us to inspect the likelihood of spurious extrapolation. Plotted are 90 random spectra (various colors) overlaid with extrapolated versions (in red) from across a dataset containing bird plumage (n = 965, from [1]) and plant reflectance spectra (n = 2350, from [2]). Colored lines indicate approximate appearance to humans. In most cases, extrapolated values seem to be reasonable, but there are some clear errors. As noted in the main text, future work should investigate the best approach for extrapolation, as well as the necessity/influence that this peripheral range of wavelengths might have on future analyses. The data underlying this figure can be found at https://doi.org/10.5061/dryad.j0zpc86nf.

Consequences of low spectral resolution

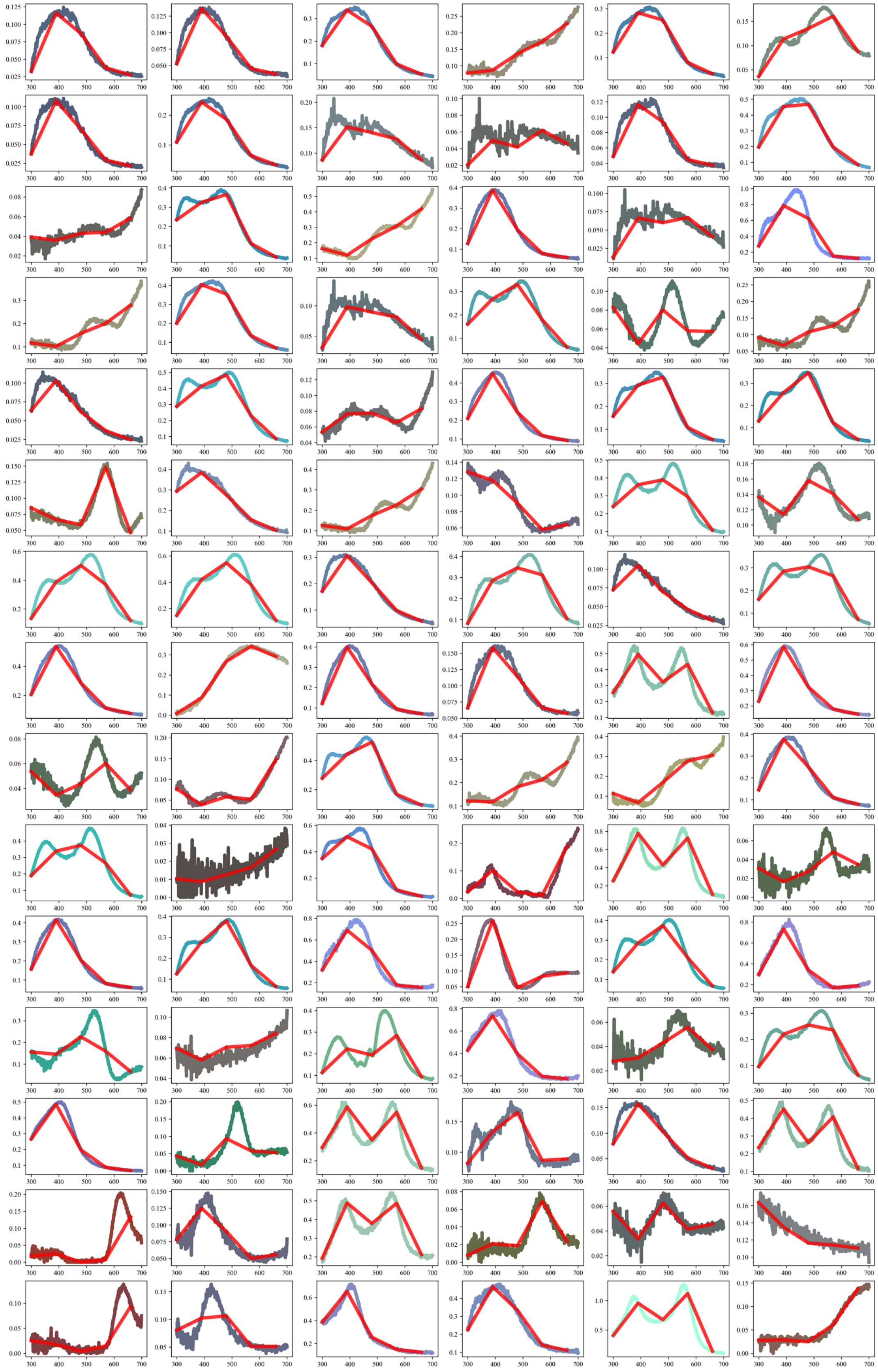

Figure D. Consequences of low spectral resolution in natural reflectance spectra. To demonstrate in principle the loss of information when reducing a full high-resolution spectrum to only 5 measurements, we overlay a set of natural reflectance spectra (colored lines) with an interpolation at only 5 wavelengths (red lines: interpolating values at 300, 390, 480, 570 and 660nm). This plot contains the 90 worst fitting interpolations (as measured by the maximum absolute difference between the red interpolations and the original data) across a dataset containing bird plumage (n = 965, from [1]) and plant reflectance spectra (n = 2350, from [2]). Colored lines indicate approximate appearance to humans. The data underlying this figure can be found at https://doi.org/10.5061/dryad.j0zpc86nf.

Embedding sampled spectra into a KLPD visual system-independent color space

Here we plot versions of the main text Figure 5 using Kullback–Leibler pseudo-divergence (KLPD: [3–5]) as a metric to measure similarity/distance between all sampled spectra. There are a variety spectral distance metrics available other than the PCA approach used in the main text including Euclidean distance, cumulative Euclidean distance and spectral angle mapping. However, these tend to be limited by conflation of brightness and spectral shape (color) differences, and by saturation of distances with increasing spectral distance [3,4]. KLPD treats reflectance spectra instead as the combination of a brightness component (dW) and a spectral shape component (dSh). Conceptually, dSh encodes the shape of the spectrum irrespective of brightness. The separation of dSh and dW components may represent unique advantages for animal color researchers, as these distances may be thought to roughly correspond to hue and brightness, respectively. The PCA approach taken in the main text was well suited to our aims in this paper, but future work should thoroughly investigate the benefits and drawbacks of alternative approaches to spectral distance (including KLPD distances).

In Python, we applied the KLPD algorithm to each pair of sampled reflectance spectra to generate the cells of a full pairwise distance matrix for each plumage patch. The large resultant matrices, however, were not easily interpretable. To reduce the dimensionality of the distance matrices, we used Uniform Manifold Approximation and Projection, (UMAP: [6], see below and main text) in order to embed spectra in a lower dimensional space that attempts to maintain distances between spectra. Spectra that fall close together in this space will tend to be those with low spectral distances, and those far apart will tend to have high spectral distances.

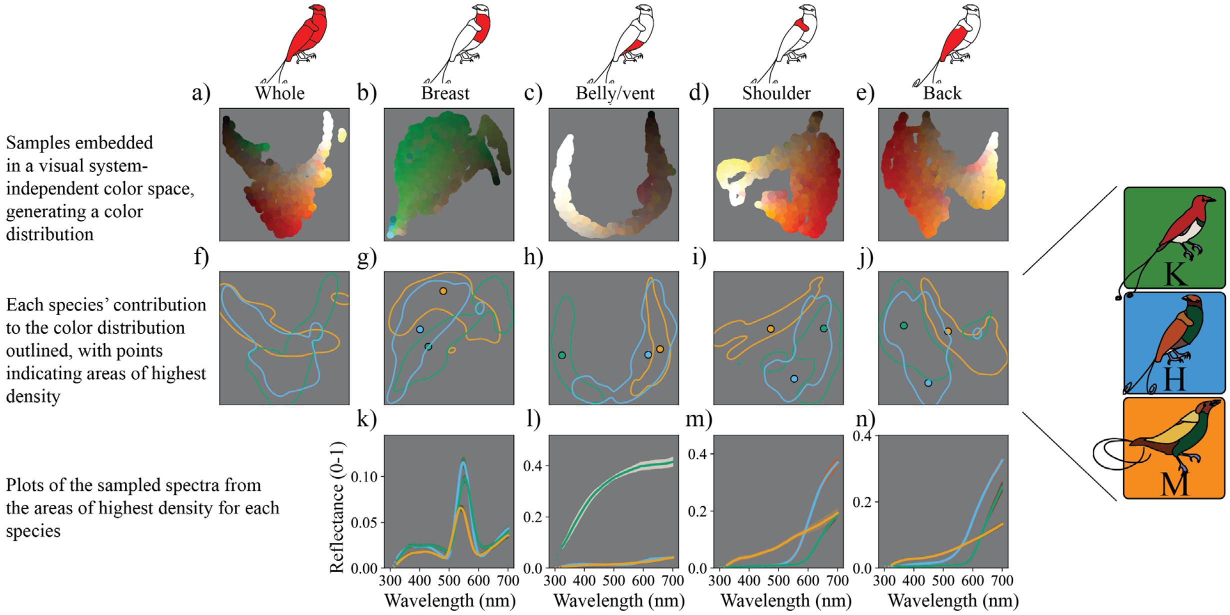

Figure E. Version of main text Fig 5, here UMAP embedding is applied to the combined brightness (dW) and shape (dSh) components of KLPD. Top row: icons indicate the patch represented in each column of the figure. (A-E) Plot of low-dimensional embedding (UMAP embedding of dSh+dW) of the sample reflectance spectra, colored according to their appearance (to human eyes). Note that the embeddings were computed separately for each patch; each column shows a different color space so should not be directly compared. (F-J) Kernel density contour plots indicating the 80% density contribution of each species to the color spaces in A-E. Line and point colors correspond to the different species as indicated in the key on the right of the figure. Areas of overlap indicate that the colors from more than one species occupy the enclosed area of color space. Points indicate the area of highest kernel density for each of the species: these areas represent the most common spectra for each species for that patch. (K-N) Plot of the 50 closest (in embedded space) spectra to the areas of highest kernel density for each patch and species (points in F-J). Bold line indicates median, shaded areas indicate median ± the median absolute deviation, and colors represent approximate color (to human eyes). The data underlying this figure can be found at https://doi.org/10.5061/dryad.j0zpc86nf.

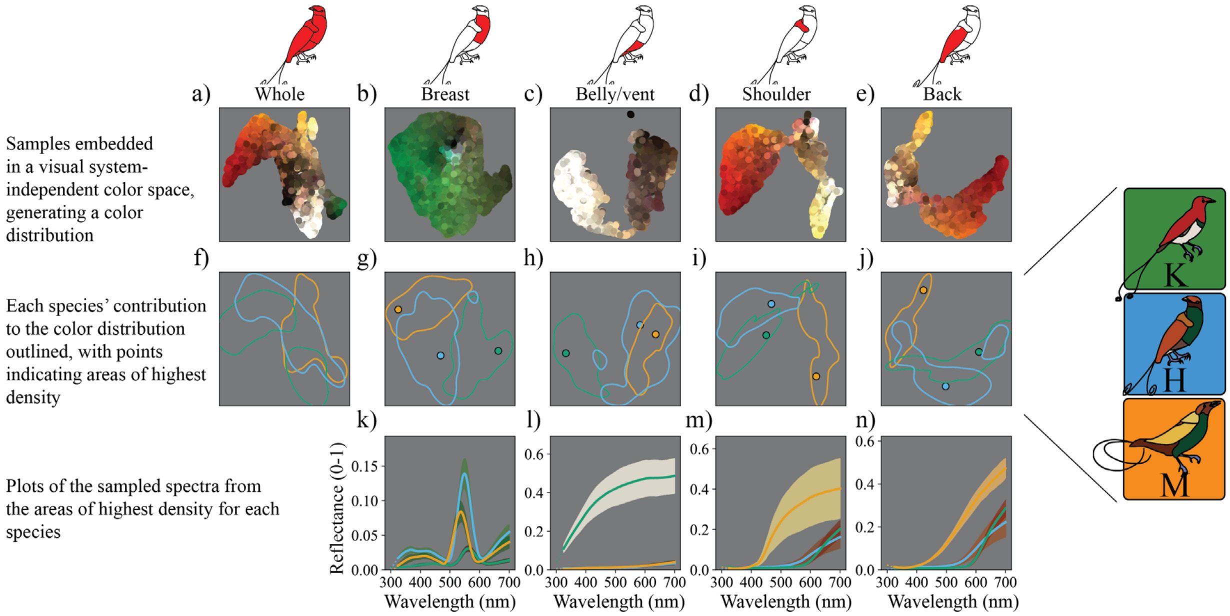

Figure F. Version of main text Fig 5, here UMAP embedding is applied to only the shape (dSh) component of KLPD. Top row: icons indicate the patch represented in each column of the figure. (A-E) Plot of low-dimensional embedding (UMAP embedding of dSh) of the sample reflectance spectra, colored according to their appearance (to human eyes). Note that the embeddings were computed separately for each patch; each column shows a different color space so should not be directly compared. (F-J) Kernel density contour plots indicating the 80% density contribution of each species to the color spaces in A-E. Line and point colors correspond to the different species as indicated in the key on the right of the figure. Areas of overlap indicate that the colors from more than one species occupy the enclosed area of color space. Points indicate the area of highest kernel density for each of the species: these areas represent the most common spectra for each species for that patch. (K-N) Plot of the 50 closest (in embedded space) spectra to the areas of highest kernel density for each patch and species (points in F-J). Bold line indicates median, shaded areas indicate median ± the median absolute deviation, and colors represent approximate color (to human eyes). The data underlying this figure can be found at https://doi.org/10.5061/dryad.j0zpc86nf.

UMAP parameterization

In the main text, we generate PCA coordinates (20 dimensions) for all sampled spectra and then we embedded these coordinates into lower dimensional spaces for interpretability. We chose to embed into two dimensions using Uniform Manifold Approximation and Projection (UMAP: [6] available from www.umap-learn.readthedocs.io), a general purpose manifold learning and dimensionality reduction approach. UMAP has two basic parameters, n_neighbors and min_dist, which determine the balance of global and local distance information in the embedding, and the minimum distance in embedded space allowed between pairs of points, respectively. For the main analysis we chose to use the default values of 15 and 0.1 for these parameters, respectively. We found qualitatively similar results using other values for these parameters (see Figs G and H below).

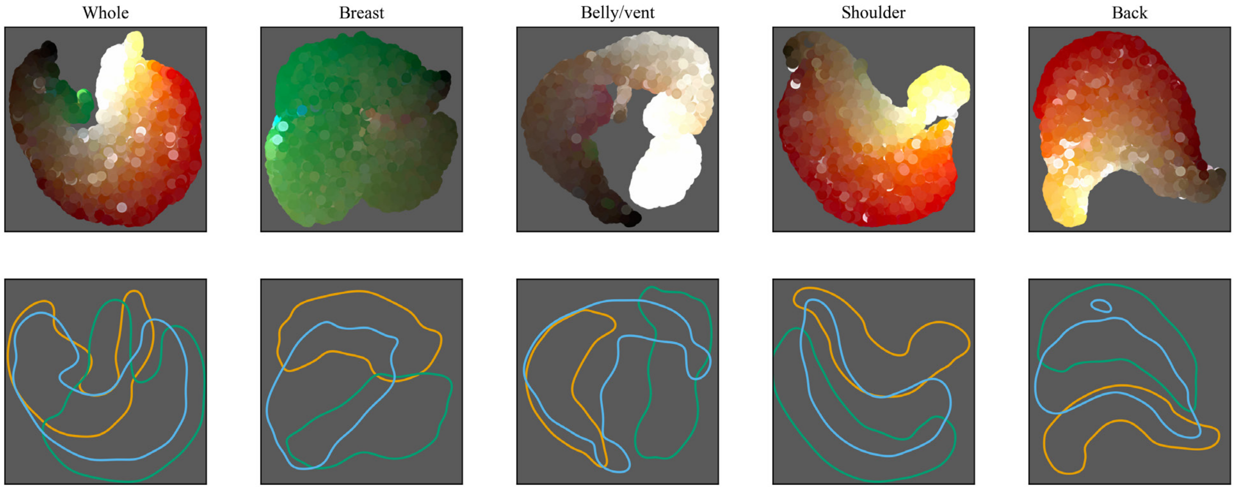

Figure G. Visual system-independent embedding of sampled spectra for each patch, using alternative settings for UMAP embedding (minimum distance = 1, versus 0.1 in the main text). Top row: plot of low-dimensional embedding (UMAP embedding of PCA) of the sample reflectance spectra, colored according to their appearance (to human eyes). Note that the embeddings were computed separately for each patch; each column shows a different color space so should not be directly compared. Bottom row: kernel density contour plots indicating the 80% density contribution of each species to the color spaces in the top row. Line colors correspond to the different species as indicated in the keys in the main text (Orange: Magnificent bird-of-paradise, Green: King bird-of-paradise, Blue: hybrid King of Holland's bird-of-paradise). Areas of overlap indicate that the colors from more than one species occupy the enclosed area of color space. The data underlying this figure can be found at https://doi.org/10.5061/dryad.j0zpc86nf.

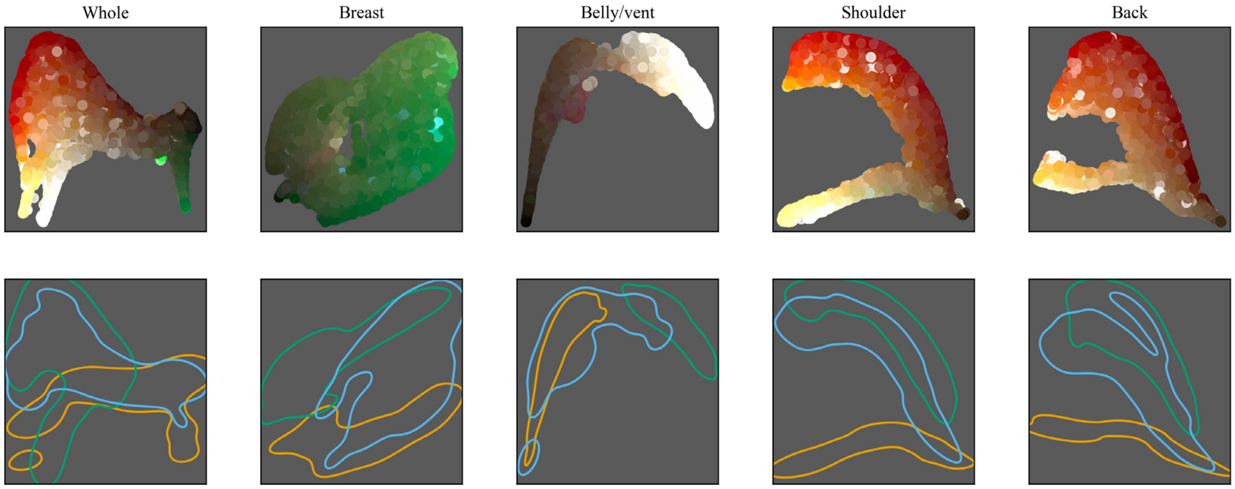

Figure H. Visual system-independent embedding of sampled spectra for each patch, using alternative settings for UMAP embedding (number of neighbors= 2000, versus 15 in the main text). Top row: plot of low-dimensional embedding (UMAP embedding of PCA) of the sample reflectance spectra, colored according to their appearance (to human eyes). Note that the embeddings were computed separately for each patch; each column shows a different color space so should not be directly compared. Bottom row: kernel density contour plots indicating the 80% density contribution of each species to the color spaces in the top row. Line colors correspond to the different species as indicated in the keys in the main text (Orange: Magnificent bird-of-paradise, Green: King bird-of-paradise, Blue: hybrid King of Holland's bird-of-paradise). Areas of overlap indicate that the colors from more than one species occupy the enclosed area of color space. The data underlying this figure can be found at https://doi.org/10.5061/dryad.j0zpc86nf.

Embedding sampled spectra into avian & human visual models

A typical approach in avian vision research is to embed (or map) spectra in the tetrahedral avian color space model. We replicated this approach, using visual pigment sensitivities for an average violet-sensitive bird (extracted from pavo [7]), with uniform lighting (irradiance) and background color, to generate relative cone-catch values, which can be converted easily into coordinates in the tetrahedral color space model [8]. Similarly, spectra were embedded into several human vision color spaces (CIExyY, sRGB) using Python package colour (0.4.3, available from www.colour-science.org). In these cases, we first converted spectra to standard XYZ tristimulus values (using CIE 1931 2 Degree Standard Observer values extrapolated to the full range of the data) and then converted into the spaces of interest. For figures in the main text, sRGB values were brightened (300%) for visibility.

Generating and comparing chromatic and achromatic color contrasts

We tested whether the patches on the King, Magnificent, and hybrid King of Holland's bird-of-paradise might be discriminable using models of avian vision and color discrimination [8–10]. For simplicity, we used the median of all sample spectra for each patch for each specimen. We then applied, using R package pavo [7], a model of avian vision similar to the one used in the main paper (average violet-sensitive avian visual pigment sensitivities, Blackbird (*Turdus merula*) ocular media transmission, Chicken (*Gallus gallus*) double-cone sensitivity, and ideal illumination and background color. Note that Chicken and Blackbird are both violet-sensitive birds [11]). We then computed both chromatic (dS) and achromatic (dL) color contrasts using default parameters (*Columba livia* cone ratios of 1:1:1:2 [10], and Weber fractions of 0.1; see [12] for discussion of the importance of these parameters). We then use the command bootcoldist to generate mean and 95% confidence intervals for the contrasts between patches. We considered those patches with a mean color contrast between groups above 3 as likely to be discriminable and below 3 as unlikely to be discriminable.

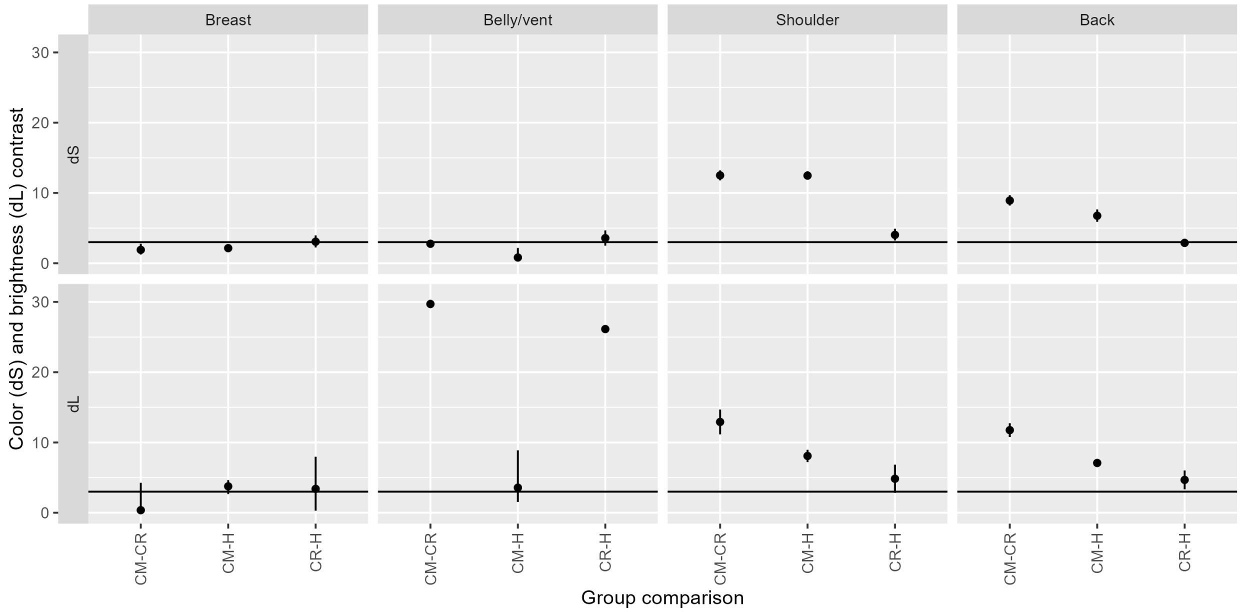

Figure I. Plot of the chromatic (dS) and achromatic (dL) contrasts between the median spectrum from each patch of the specimens. Mean values plotted as points, and 95% confidence intervals are plotted as bars. Shown are comparisons between the Magnificent bird-of-paradise (CM), King bird-of-paradise (CR), and hybrid King of Holland's bird-of-paradise (H). Where both mean and 95% confidence intervals fall above a color contrast value of 3, we consider the two colors to be discriminable. We generated these estimates using visual sensitivities, cone densities and Weber fractions as described in the 'Generating and comparing chromatic and achromatic color contrasts' section). The data underlying this figure can be found at https://doi.org/10.5061/dryad.j0zpc86nf.

Phylogeny

Table B. Table of GenBank accession codes for genes for each specimen and species found in the subfamily Paradisaeinae.

Species	co1	cytB	g3pdh	ODC
Astrapia mayeri		GQ334231	GQ334303	GQ334268
Astrapia nigra		GQ334228	GQ334300	GQ334265
Astrapia rothschildi		GQ334232	GQ334304	GQ334269
Astrapia splendidissima		GQ334229	GQ334301	GQ334266
Astrapia stephaniae		GQ334230	GQ334302	GQ334267
Cicinnurus regius		GQ334249	GQ334320	GQ334285
Cicinnurus regius	AF197831	U15201		
Diphyllodes magnificus		GQ334247	GQ334318	GQ334283
Diphyllodes magnificus		X74255.1		
Diphyllodes magnificus		EF592218.1		
Diphyllodes magnificus		AF308772		
Diphyllodes respublica		GQ334248	GQ334319	GQ334284
Diphyllodes respublica	AF197830	U15200		
Epimachus fastuosus		GQ334244	GQ334315	GQ334280
Epimachus fastuosus		X74253		
Epimachus meyeri		U15206		
Epimachus meyeri		GQ334245	GQ334316	GQ334281
Lycocorax pyrrhopterus		GQ334221	GQ334294	GQ334259
Paradigalla brevicauda		GQ334227	GQ334299	GQ334264
Paradigalla carunculata		GQ334226	GQ334298	GQ334263
Paradisaea (raggiana) augustaevictoriae	AF197828	U25738		
Paradisaea (raggiana) augustaevictoriae		U15204.1		
Paradisaea apoda		GQ334252	GQ334323	GQ334288
Paradisaea decora		GQ334256		
Paradisaea guilielmi		GQ334257	GQ334327	GQ334292
Paradisaea minor		GQ334254	GQ334325	GQ334290
Paradisaea minor		U25737		
Paradisaea raggiana		GQ334253	GQ334324	GQ334289
Paradisaea raggiana		EF592241		
Paradisaea raggiana		AF308774		
Paradisaea rubra		GQ334255	GQ334326	GQ334291
Paradisaea rubra	AF197829	U25736		
Paradisaea rudolphi		GQ334258	GQ334328	GQ334293
Paradisaea rudolphi		U15203		
Phonygammus keraudrenii	AF197826	X74252		
Pteridophora alberti		GQ334238	GQ334310	GQ334275
Seleucidis melanoleucus		GQ334251	GQ334322	GQ334287
Seleucidis melanoleucus		U15202		

We generated alignments for each gene using MUSCLE in Geneious Prime 2022.0.1 (www.geneious.com/) before running Bayesian phylogenetic reconstruction in BEAST v2.6.6 [13], with data partitioned by gene (and codon position for the mitochondrial genes). Each partition had independent averaged site models generated with bModelTest [14]. Partitions shared a strict clock, used a birth death model, and default priors were used throughout. This model was run for one chain of 200 million samples, with a burn-in of 20 million samples, with trees logged every 200 thousand samples, resulting in 1000 trees. The lowest resultant effective sample size was 281. We then used TreeAnnotator to generate an MCMC maximum tree credibility consensus tree (see Fig J), which we then simplified for main text Fig 2. The topology is identical to that of a more comprehensive genetic reconstruction of this clade [15]. We produced our own phylogeny here in order to add several specimens and the co1 gene into the analysis, as well as to allow us to accurately generate the topology for main text Fig 2.

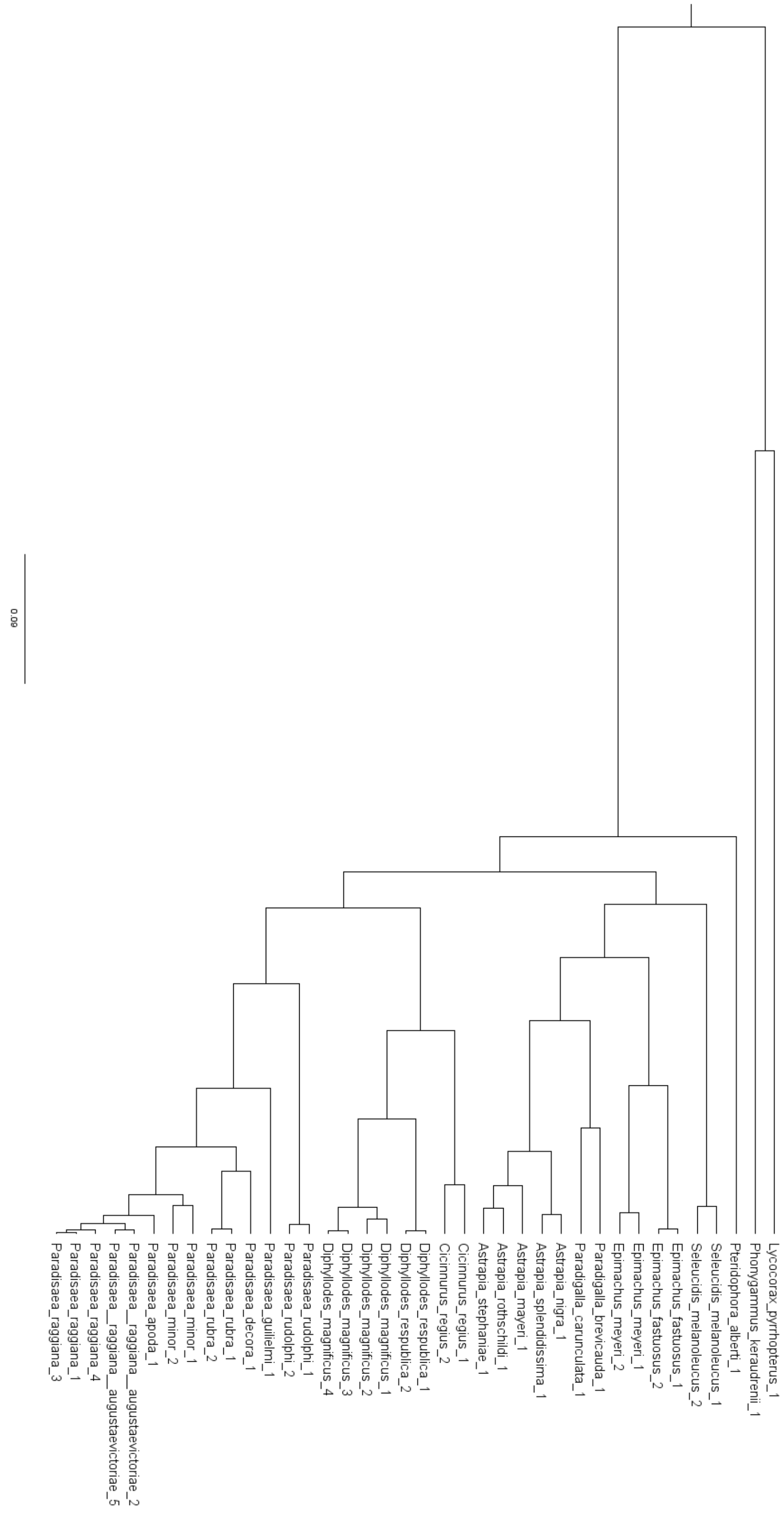

Figure J. Bayesian maximum likelihood consensus tree from which main text Fig 2 was generated, showing all specimens for each species and with all genera expanded.

Sampling and patches

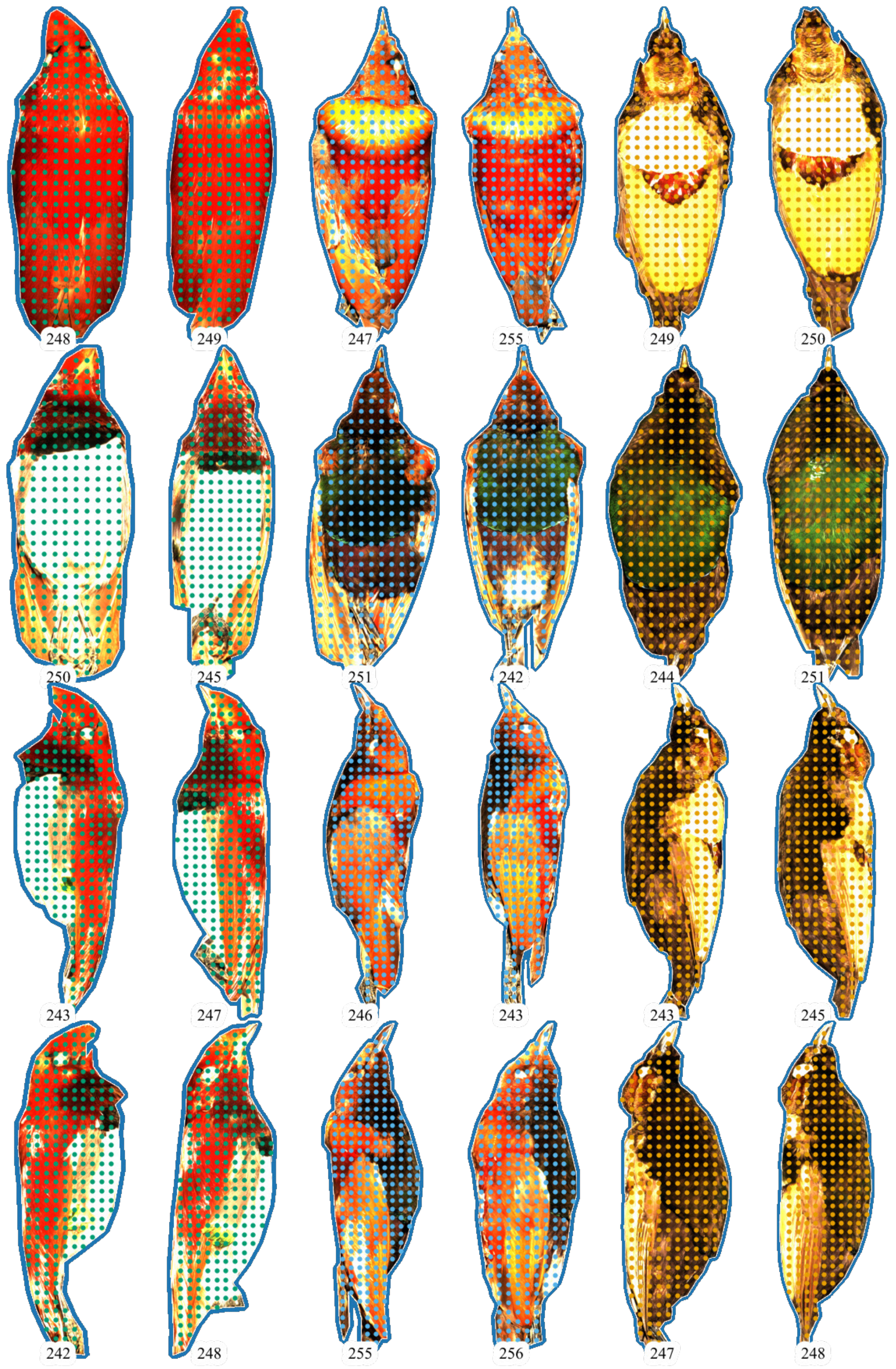

Figure K. Images indicating the location of samples for each of the 'Whole' body plans in the main paper. Rows of images show alternate views of each of the six specimens (each column is a unique specimen), the blue outline indicates the extent of the region of interest for the given image, and points indicate sampled locations. The number below each image indicates the number of samples extracted from the pictured specimen and view.

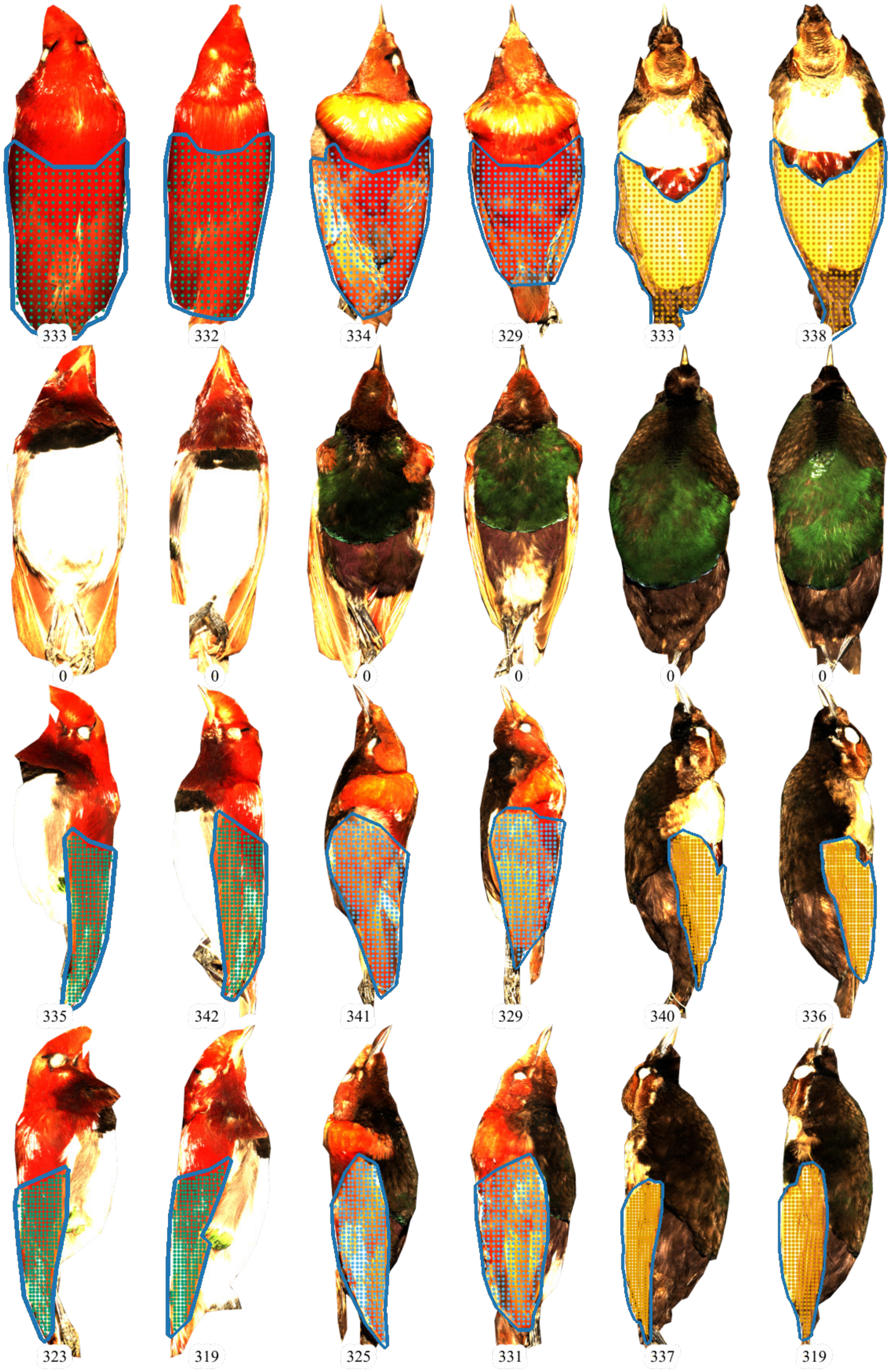

Figure L. Images indicating the location of samples for each of the ‘Back’ patches in the main paper. Rows of images show alternate views of each of the six specimens (each column is a unique specimen), the blue outline indicates the extent of the patch region of interest for the given image, and points indicate sampled locations. The number below each image indicates the number of samples extracted from the pictured specimen and view.

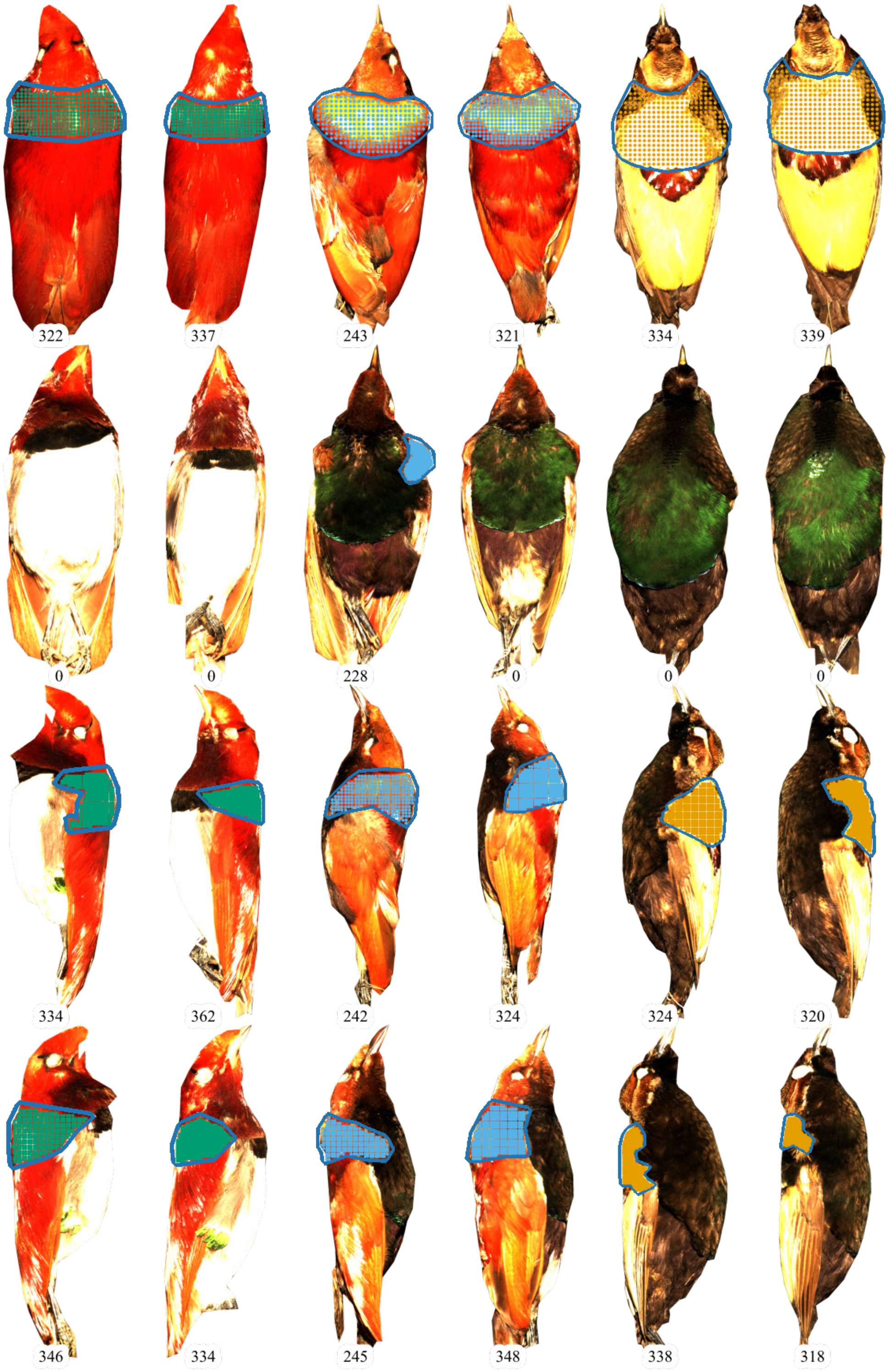

Figure M. Images indicating the location of samples for each of the 'Shoulder' patches in the main paper. Rows of images show alternate views of each of the six specimens (each column is a unique specimen), the blue outline indicates the extent of the patch region of interest for the given image, and points indicate sampled locations. The number below each image indicates the number of samples extracted from the pictured specimen and view.

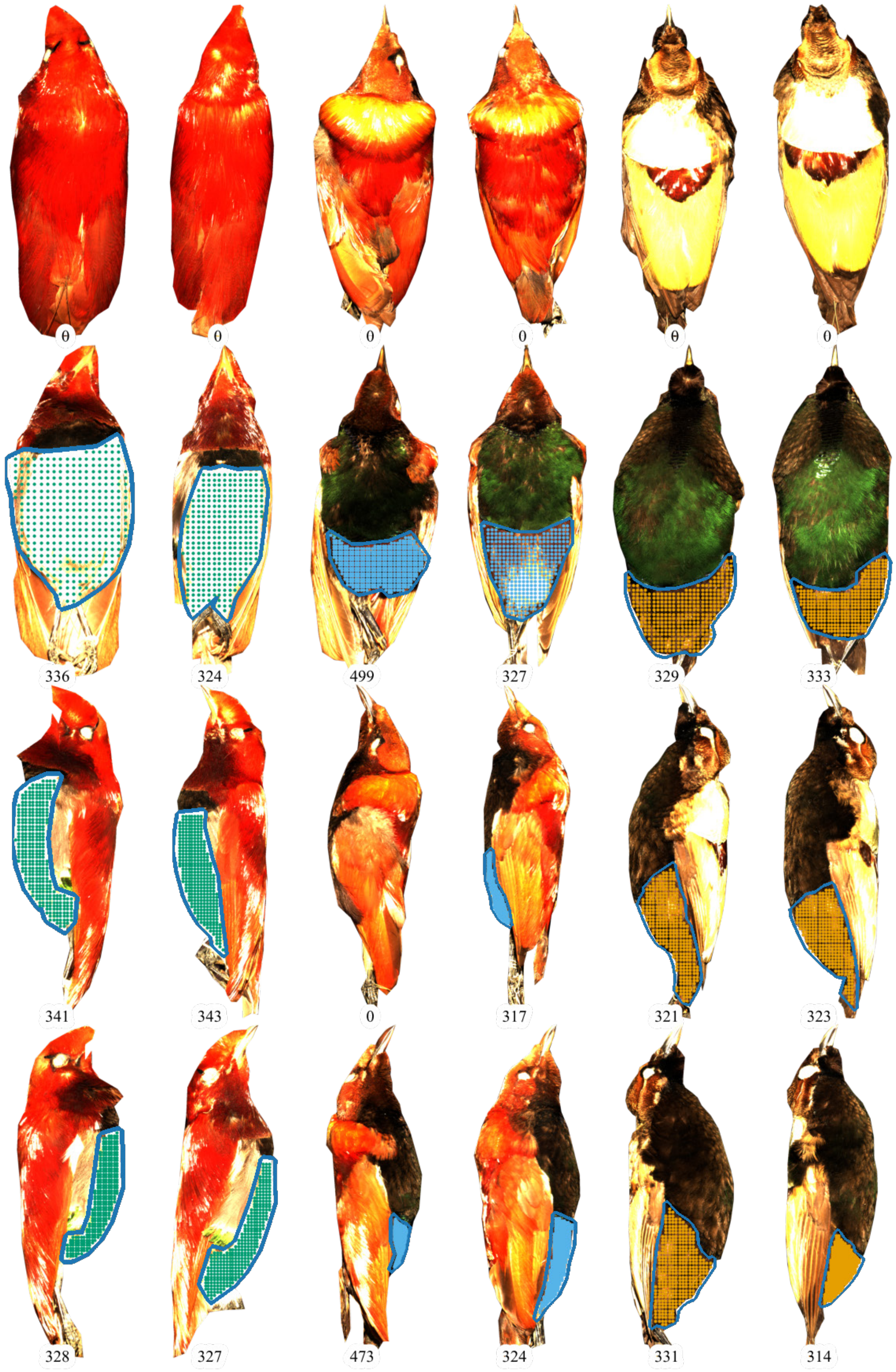

Figure N. Images indicating the location of samples for each of the 'Belly' patches in the main paper. Rows of images show alternate views of each of the six specimens (each column is a unique specimen), the blue outline indicates the extent of the patch region of interest for the given image, and points indicate sampled locations. The number below each image indicates the number of samples extracted from the pictured specimen and view.

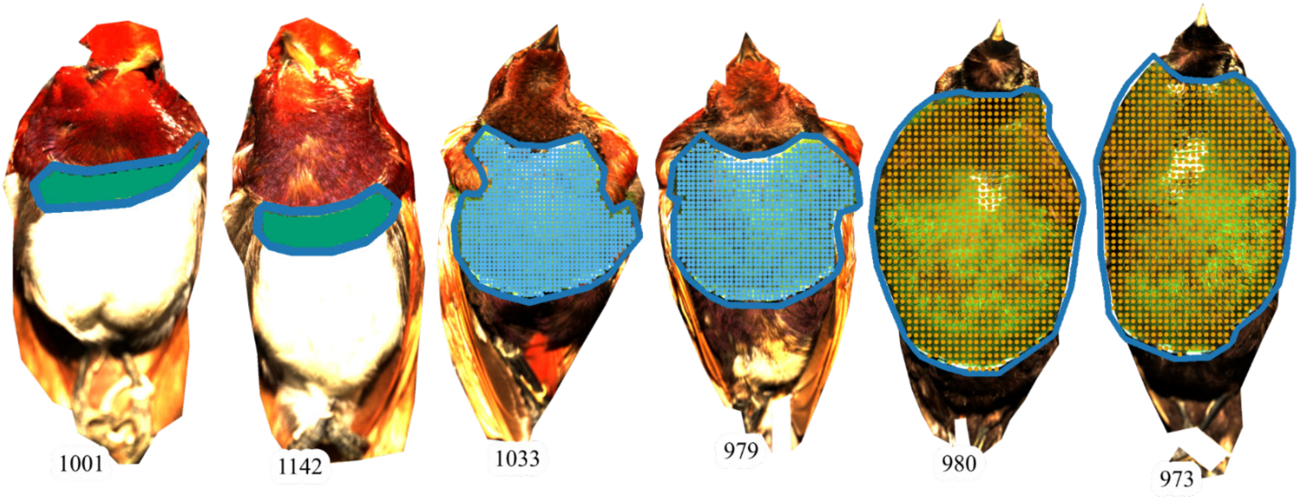

Figure O. Images indicating the location of samples for each of the ‘Breast’ patches in the main paper. Each image shows a unique specimen, the blue outline indicates the extent of the patch region of interest for the given image, and points indicate sampled locations. The number below each image indicates the number of samples extracted from the pictured specimen and view.

Additional plots

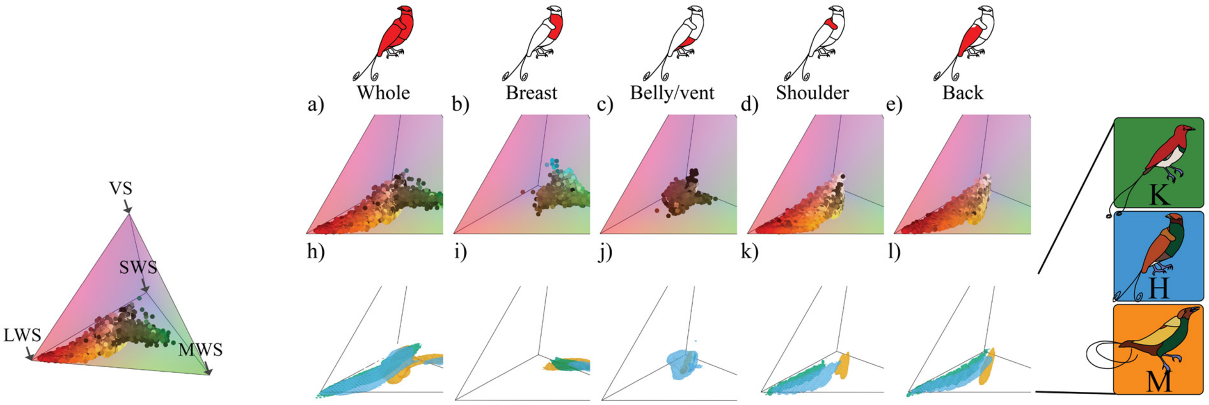

Figure P. Version of main text Fig 6, where the z-axis of the tetrahedral avian color space has not been collapsed to 2D. Here we plot an oblique view of the avian color space tetrahedron. Extreme left: a labeled 3D tetrahedron with vertex labels indicating the relevant photoreceptor stimulation. V: Violet wavelength-sensitive, S: Short wavelength-sensitive, M: Medium wavelength-sensitive, and L: Long wavelength-sensitive. In each of the other panels, we zoom in on the section of this tetrahedron containing sampled colors. In the upper row: points in this space indicate sampled spectra from the 'Whole', 'Breast', 'Belly/vent', 'Shoulder' and 'Back' patches across all specimens, respectively. Points are colored according to their appearance (to human eyes). In the lower row: 3D kernel density plots show the 95% density for each species' sampled points for each patch. Colors of the kernel densities indicate species as indicated in the species key on the extreme right; orange: Magnificent bird-of-paradise, green: King bird-of-paradise, blue: hybrid King of Holland's bird-of-paradise. The data underlying this figure can be found at https://doi.org/10.5061/dryad.j0zpc86nf.

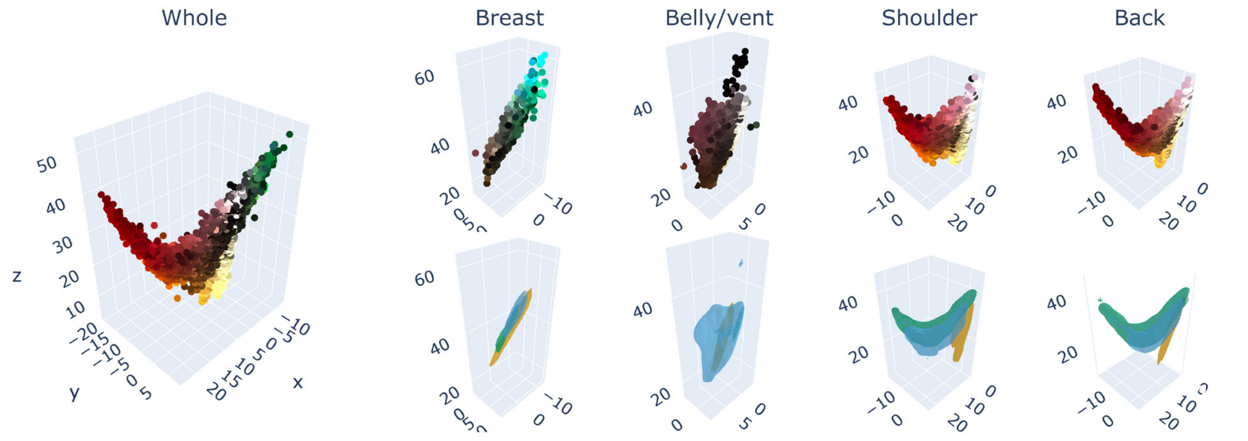

Figure Q. Visual system-dependent embedding of sampled spectra for each patch, using the receptor noise limited (RNL) color space (as defined in [16]), using visual sensitivities, cone densities and Weber fractions as described in the 'Generating and comparing chromatic and achromatic color contrasts' section). In the RNL color space, the axes represent color distance as computed by modeled opponency mechanisms (x: MWS:LWS, y: MWS+LWS:SWS, z: MWS+LWS+SWS:VS) and distance between points indicates discriminability. The Euclidean distance between points is similar to dS color contrasts as described in the 'Generating and comparing chromatic and achromatic color contrasts' section; points that are more than 3 units apart might be considered discriminable. The panel to the left shows sampled spectra for the 'Whole' patch colored by approximate appearance to humans. The other panels show, from left to right the 'Breast', 'Belly/vent', 'Shoulder', and 'Back' patches, respectively. The upper row shows points colored by approximate appearance to humans, and the lower row indicates the 3D kernel density (95% density) for each species' sampled colors for each patch. Colors of the kernel densities indicate species as indicated in the species key in the main text; orange: Magnificent bird-of-paradise, green: King bird-of-paradise, blue: hybrid King of Holland's bird-of-paradise. The plotted distributions indicate that when all of the samples are considered, each patch from each species probably contains many discriminable colors. The data underlying this figure can be found at https://doi.org/10.5061/dryad.j0zpc86nf.

3D Model

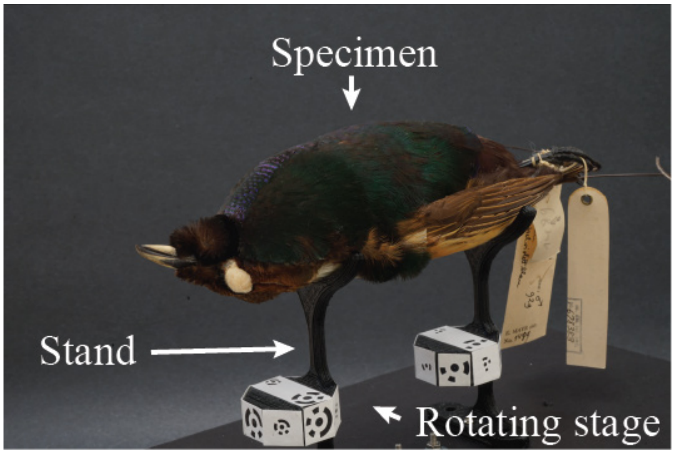

Figure R. Diagram of the set-up used during photogrammetry, showing the specimen resting on a custom designed stand with calibration targets, mounted onto a rotating stage. Note that the coded targets shown on each leg of the stand were not used in generating the alignment.

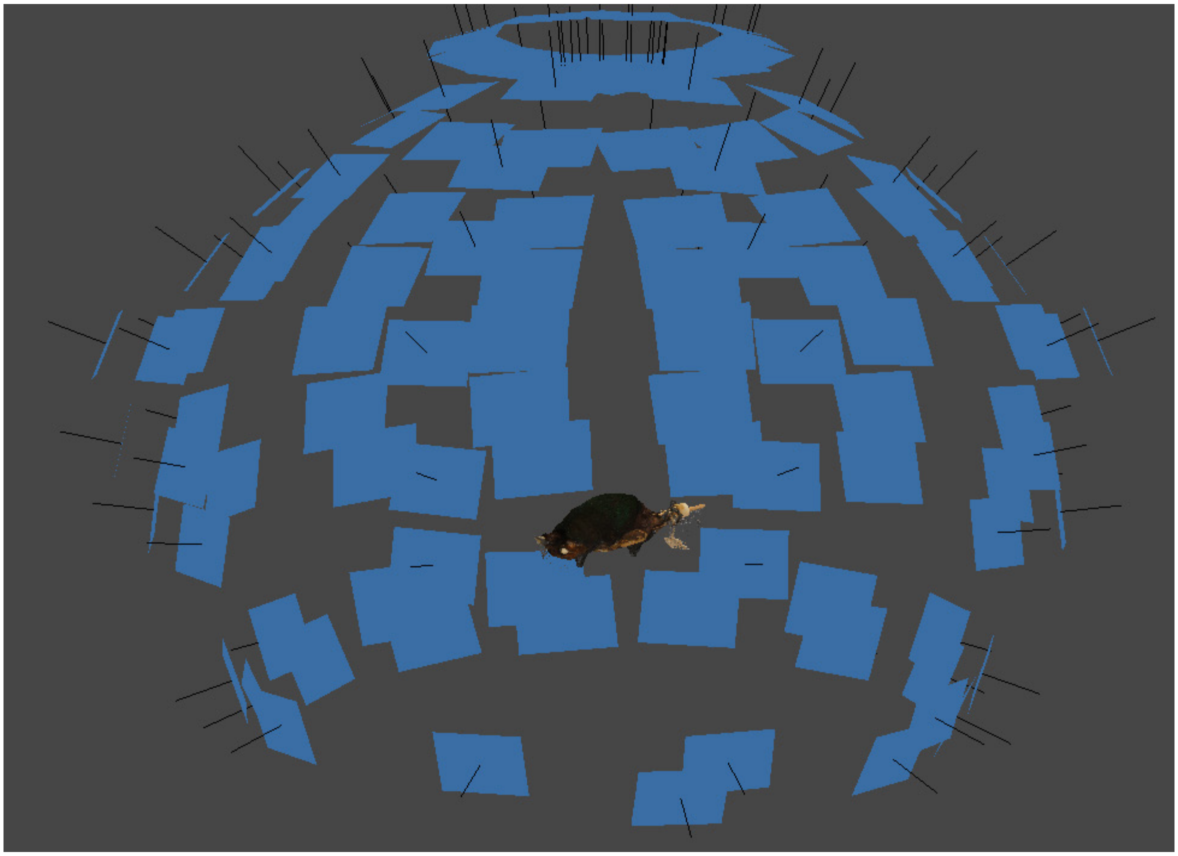

Figure S. Screen capture from Metashape (v2.0.2, Agisoft LLC, Saint Petersburg, Russia). In the center, we show one constructed 3D model, textured with information from photographs. Each blue rectangle indicates the computed position of the camera relative to the sample. Black lines indicate the normal of the center of each photograph. This image shows the entire set of photographs, and the computed model for the ventral view of one specimen as seen in Fig 4 in the main text.

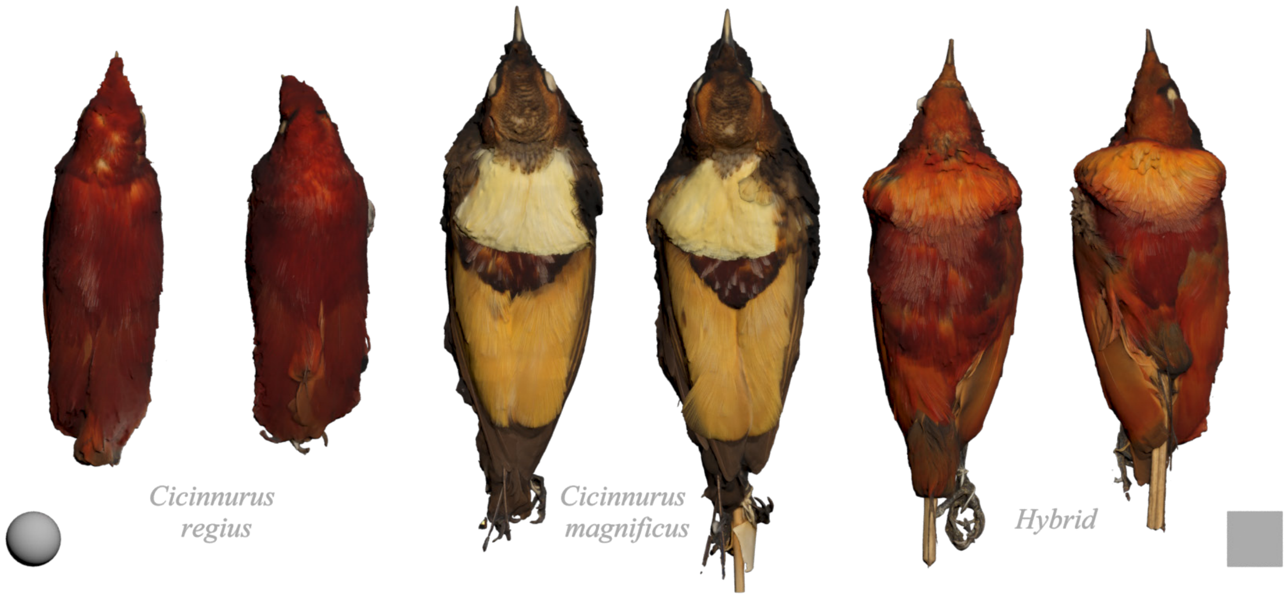

Figure T. Renders of the 3D models for each specimen, showing the dorsal side. Models are to scale: the gray square in the bottom right has a height and width of 2cm. See also S1 Video.

In order to relate surface angle information from the 3D models to the hyperspectral images, registration between the two was required. We imported false-color RGB versions of our hyperspectral images into the whole-specimen alignments generated in Agisoft Metashape (see Fig S). We then marked on each hyperspectral image points of known location (tip of beak, edge of eye, tip of left wing, etc.), which allowed us to roughly align these images to the 3D model; i.e., to calculate the location and angle from which the hyperspectral image was captured (termed camera extrinsics) in relation to the 3D model. We describe this approach as rough because the images captured from (push-broom) hyperspectral cameras have some unique properties. One is that the dimensions of the hyperspectral image are limited in one dimension (width), but unlimited in the other (length) because images are made by repeated exposures of a line of fixed length (width) while moving the specimen through the other dimension (height). Essentially the user controls the height of the image. This panning capture also means that lens distortion is limited to one axis of the image. This means that for push-broom cameras the computations that allow for 3D models to be generated from these distorted images (termed camera intrinsic models) are non-standard, and may differ per image. The approach also relies on a small number of manually designated points, which might not necessarily include enough information for a very accurate calculation of the camera extrinsics. These factors together result in sub-optimal registration and future work should address this.

With registration between our hyperspectral images and 3D models, it was possible to render the 3D models from the point of view of the hyperspectral imager. For the main text, we rendered the surface normal of the model in relation to the hyperspectral imager. This allows us to know the surface angle of the specimen for any given hyperspectral sample. Our goal was to get a better idea of how the effective reflectance of the iridescent breast varied with vertical and horizontal angle relative to the imager. We then generated plots of breast color against both of these angles (with samples taken from all images of all specimens that contain the breast patch, see main text Fig 4).

Tail morphology model

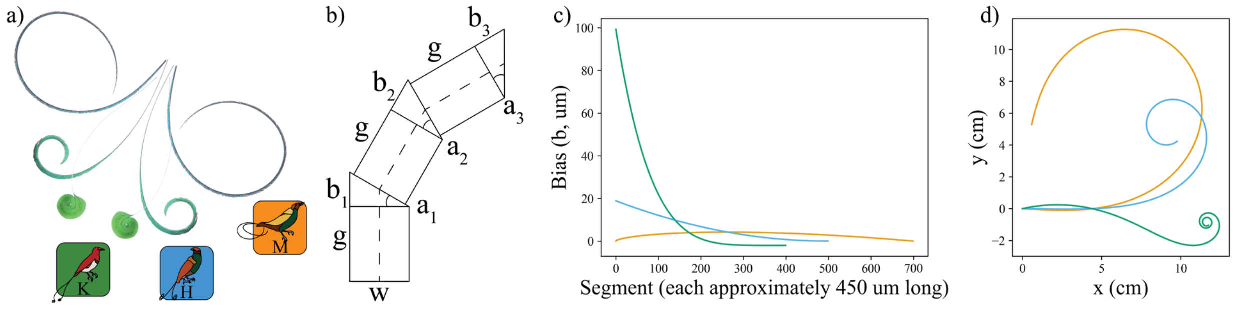

Figure U. Analyzing the shapes of bird-of-paradise tails. Modified tail feathers of the hybrid King of Holland's bird-of-paradise appear to be morphologically intermediate to those of the King and Magnificent bird-of-paradise. (A) Overlaid photographs of the dorsal side of the central modified tail feathers from each of the species, as indicated by the location of the species icons. Photos: David Ocampo. (B) Diagram of the simple model of the development of tail feathers. Segments (3 shown) are sequentially attached to the leading edge of the previous segment, building up a curved shape. Each segment has a width (w), a length (g), and additional amount of growth biased to one side of the segment (b), generating a leading edge with angle (a). Segment bias (b) is controlled as a function of segment number, and the resulting angle (a) is recorded to reproduce rachis shape. (C) Graph showing the parameters responsible for generating shapes shown in (D). Here the bias in the length of the side of the segments is plotted as a function of number of segments attached as the feather rachis develops. (D) Rachis shapes produced by the model of tail development, with parameters manually chosen to approximate the tail feathers of the King, Magnificent and hybrid birds-of-paradise (A).

We found that a simple developmental model allowed us to generate rachis shapes similar to those of the modified tail feathers (sometimes called tail wires) of the King, Magnificent and hybrid King of Holland's bird-of-paradise. For simplicity, we assumed that each segment of the growing rachis had width (w) and length (g) of 450 µm, based on a rough estimate from a photograph containing a scale bar. Varying both i) the bias of segments as a function of the number of segments; and ii) the total number of segments allowed us to replicate the shapes and sizes of the King, Magnificent, and hybrid (Fig U-A, D). The King bird-of-paradise rachis can be modeled in 400 sections; it grows with a strong bias on one side of the rachis with the bias reducing as the feather grows. When the rachis has grown to ¾ of its final length the bias is slightly negative (the growth is biased toward the opposite side of the rachis, Fig U-C). This profile generates the tightly curled distal spatula, and the slightly recurved base of the rachis (Fig U-A, C). The Magnificent bird-of-paradise rachis can be modeled in 800 segments, with little bias at the start and end of growth, and only low bias in between (Fig U-C). This generates the relatively straight proximal and distal ends of the rachis with a gentle arc in-between (Fig U-A, D). The hybrid rachis is modeled as comprising 500 segments, with a profile of bias intermediate to the King and Magnificent (Fig U-A, C, D). While our simple model only considers rachis shape, the hybrid tail appears to be morphologically intermediate in other ways. Only the distal spatula of the King bird-of-paradise tail feather contains barbs, whereas the whole length of the Magnificent tail feather contains barbs. The hybrid appears to be intermediate here too, with barbs present on approximately ¼ of the length of the rachis, though in all cases barbs are apparent only on the outside edge of the rachis (Fig U-A).

Fluorescence

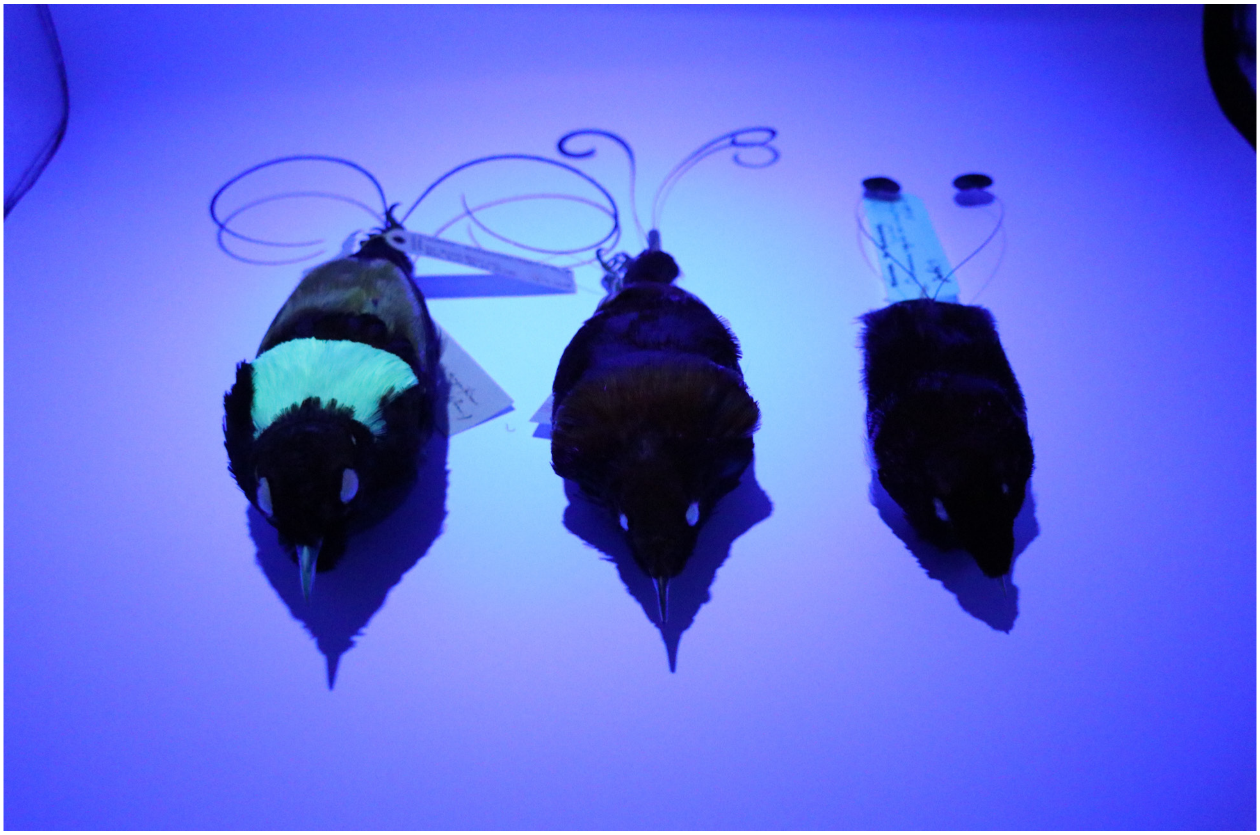

Figure V. On the advice of Glenn Seeholzer, we photographed specimens under a small blacklight torch, (390nm - 395nm emission). The Magnificent bird-of-paradise's shoulder/mantle patch fluoresces a bright off-white under this lighting. Interestingly, no appreciable fluorescence is seen on the homologous patches on the hybrid King of Holland's bird-of-paradise, or King bird-of-paradise. Future work could quantify and investigate the source of this fluorescence. Photo credit: David Ocampo.

References

1. Stoddard MC, Prum RO. How colorful are birds? Evolution of the avian plumage color gamut. Behav Ecol. 2011 Sep 1;22(5):1042–52.

2. Arnold SEJ, Faruq S, Savolainen V, McOwan PW, Chittka L. FReD: The Floral Reflectance Database — A Web Portal for Analyses of Flower Colour. PLOS ONE. 2010 Dec 10;5(12):e14287.

3. Deborah H, Richard N, Hardeberg JY. A comprehensive evaluation of spectral distance functions and metrics for hyperspectral image processing. IEEE J Sel Top Appl Earth Obs Remote Sens. 2015 Mar 18;8(6):3224–34.

4. Deborah H, Richard N, Hardeberg JY. Application of spectral statistics to spectral texture discrimination. In: 2018 Colour and Visual Computing Symposium (CVCS). 2018. p. 1–6.

5. Richard N, Helbert D, Olivier C, Tamisier M. Pseudo-divergence and bidimensional histogram of spectral differences for hyperspectral image processing. J Imaging Sci Technol. 2016 Oct 9;60(5):050402–1.

6. McInnes L, Healy J, Melville J. UMAP: Uniform manifold approximation and projection for dimension reduction. ArXiv180203426 Cs Stat [Internet]. 2020 Sep 17 [cited 2021 Mar 15]; Available from: http://arxiv.org/abs/1802.03426

7. Maia R, Eliason CM, Bitton P, Doucet SM, Shawkey MD, Tatem A. pavo: an R package for the analysis, visualization and organization of spectral data. Methods Ecol Evol. 2013 May 22;4(10):906–13.

8. Stoddard MC, Prum RO. Evolution of avian plumage color in a tetrahedral color space: a phylogenetic analysis of new world buntings. Am Nat. 2008 Jun;171(6):755–76.

9. Endler JA, Mielke PW. Comparing entire colour patterns as birds see them. Biol J Linn Soc. 2005 Dec 1;86(4):405–31.

10. Vorobyev M, Osorio D. Receptor noise as a determinant of colour thresholds. Proc R Soc B. 1998 Mar 7;265(1394):351–8.

11. Hart NS, Partridge JC, Cuthill IC, Bennett ATD. Visual pigments, oil droplets, ocular media and cone photoreceptor distribution in two species of passerine bird: the blue tit (Parus caeruleus L.) and the blackbird (Turdus merula L.). J Comp Physiol A. 2000 Apr 1;186(4):375–87.

12. Olsson P, Lind O, Kelber A, Simmons L. Chromatic and achromatic vision: parameter choice and limitations for reliable model predictions. Behav Ecol. 2018 Mar 14;29(2):273–82.

13. Bouckaert R, Heled J, Kühnert D, Vaughan T, Wu CH, Xie D, et al. BEAST 2: A software platform for Bayesian evolutionary analysis. Prlic A, editor. PLoS Comput Biol. 2014 Apr 10;10(4):e1003537.

14. Bouckaert R, Drummond AJ. bModelTest: Bayesian phylogenetic site model averaging and model comparison. BMC Evol Biol. 2017 Feb 6;17(1):42.

15. Irestedt M, Jønsson KA, Fjeldså J, Christidis L, Ericson PG. An unexpectedly long history of sexual selection in birds-of-paradise. BMC Evol Biol. 2009 Sep 16;9(1):235.

16. Renoult JP, Kelber A, Schaefer HM. Colour spaces in ecology and evolutionary biology. Biol Rev. 2017 Oct 15;92(1):292–315.
