# Supplementary material for: Hyperspectral imaging in animal coloration research: A user-friendly pipeline for image generation, analysis, and integration with 3D modeling
Source: PLoS Biol. 2024 Dec 3;22(12):e3002867. doi: 10.1371/journal.pbio.3002867 (PMC11614258; doi:10.1371/journal.pbio.3002867)
Supplement: Attachment — Submitted filename: Hogan and Stoddard Review.pdf [file pbio.3002867.s003.pdf]

Dear Drs. Hogan and Stoddard,

Thank you for the opportunity to review this very exciting Method and Resource manuscript. In this manuscript you propose the broader application of hyperspectral photography for quantifying museum data. The project uses a commercially available system and a workflow that you have designed, which should allow for your technique to be adopted broadly. Finally, you provide an ecologically and evolutionary meaningful example, by way of the King of Holland's bird-of-paradise. This is a nice example not only because it is visually appealing, but because it (and its parent species) provide a nice array of colors to showcase the method. I'm very supportive of your manuscript and would be eager to use it myself! Thus, I imagine there will be wide appeal among the PLoS Biology readership. What I'm most pleased by was how you described *why* such a system is useful; this technique provides users with spatially explicit color information, without compromising spectral resolution. In fact, you will see that I actually would like to see more of this. Most importantly the technique provides data that makes few assumptions. Therefore, once specimens are processed their data can be used by you, me, or any other user to ask new questions.

As you can tell, I'm very supportive of your work, so much so that I can't wait to adopt it! That being said, I do think there are areas that could be improved. I will outline these below in detail, but they broadly fall into the following categories:

1. Emphasis
2. Details on lighting
3. Highlighting potential applications
4. Clearer description of the pipeline itself

Apologies for the length of my comments for these major critiques. Hopefully these outline my points clearly. I want to avoid you needing to write lengthy responses back, so I have provided an underlined summary for each major comment. Hopefully these make it easier for you to have one response back to each major category, rather than very many responses. In addition, I have listed a few minor suggestions following these larger comments. I hope that you find my feedback useful and constructive.

Thank you,

Daniel Hanley

Major comments:

Emphasis

As a Methods and Resources contribution, I would really be looking for greater emphasis on the method and less on the interesting biological contribution. I understand that it is imperative to communicate the unique contribution your method will make for the field; however, I think that you have made that case, and it could be better integrated into the description of the method itself. While, I really love the discussion of the hybrid bird-of-paradise my overall suggestion would be to devote less space in outlining their biology (background, descriptions, implications,

etc.) and more on how your method allowed you to record data that will address these questions. In this case, I believe you have imaged 1 specimen of each species which seems sufficient to describe the method but less sufficient to fully address the biology. I feel it would be better if the emphasis could shift more to hyperspectral imagery and more specifically the workflow you designed.

Naturally, you will want to follow the recommendations of the editors on this point; however, it was something that was emphasized to our lab when we recently wrote for this outlet, and hinted at in the review invitation, “While we value the inclusion of novel biological insight in our 'Methods and Resources' articles, this is not a condition for publication of this article type in PLOS Biology.” I *loved* your biological insight, but think it could be reduced and the resource description could be expanded.

Summary: Please consider reducing the emphasis on the biological interpretation and refocus your emphasis on the contributions of your new pipeline and the practical considerations on how one might use that pipeline.

Details on lighting

Your method is tantalizing; however, there are many practical considerations that I am still unsure how I would tackle if I were to adopt this today. The largest of these has to do with controlling light while measuring your specimens. You *did* discuss what light source you were using, and importantly even described the importance of considering the effect of directional light sources on measurements and interpretation. However, I found details lacking in this regard and frankly wouldn’t be able to adopt this workflow without knowing.

For example, was your xenon arc lamp in free air in a custom fixture? If so, which type. Bulbs like this (see bulb in image) are typically in a housing (see housing in image), which provide strong directional lighting and provide adapters for various ways to cast the light. If the light was cast through optics what type were used. I imagine you may have reflected the light off of a surface and had that cast light on your subject, or used UV-VIS diffusers (e.g., from Edmund Optics).

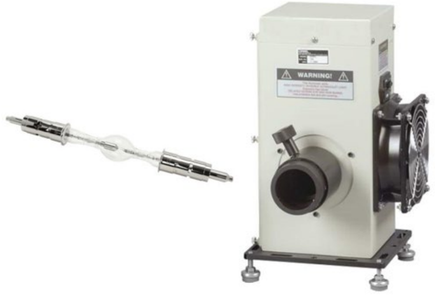

Furthermore, I would be eager to know more about where these lights were placed relative to your samples. I hate to be picky here, but these are the questions I would want answers to when I adopt your technique. I also think they are vitally important. When photographing specimens, I aim to diffuse the light as much as is possible, not because I think this replicates nature, but because our specimens are measured with reference to standards. Light from these bulbs has a distinct and predictable power spectrum that means some areas of the image will receive more light. Therefore, the same 99% Spectralon standard will not necessarily have the same value in

the top right corner as it will in the bottom left (there's a 2% shift in the image below). Naturally, if this is the case, it will impact your accuracy of the specimens as well. To account for this, I've seen some authors use full field calibrations but don't think that's necessary in your case. This tends to occur with indoor photography more than natural light, which is preferred. Below are a few illustrative images taken of standards under a free-air bulb (~1.25 m above the specimens). You also mention the impact of shape, which invariably impacts colors even if consistently colored (e.g., the Styrofoam hemisphere), and even slightly angling your Spectralon can impact it's color by up to 20% (from flat to angled at ~45 degrees).

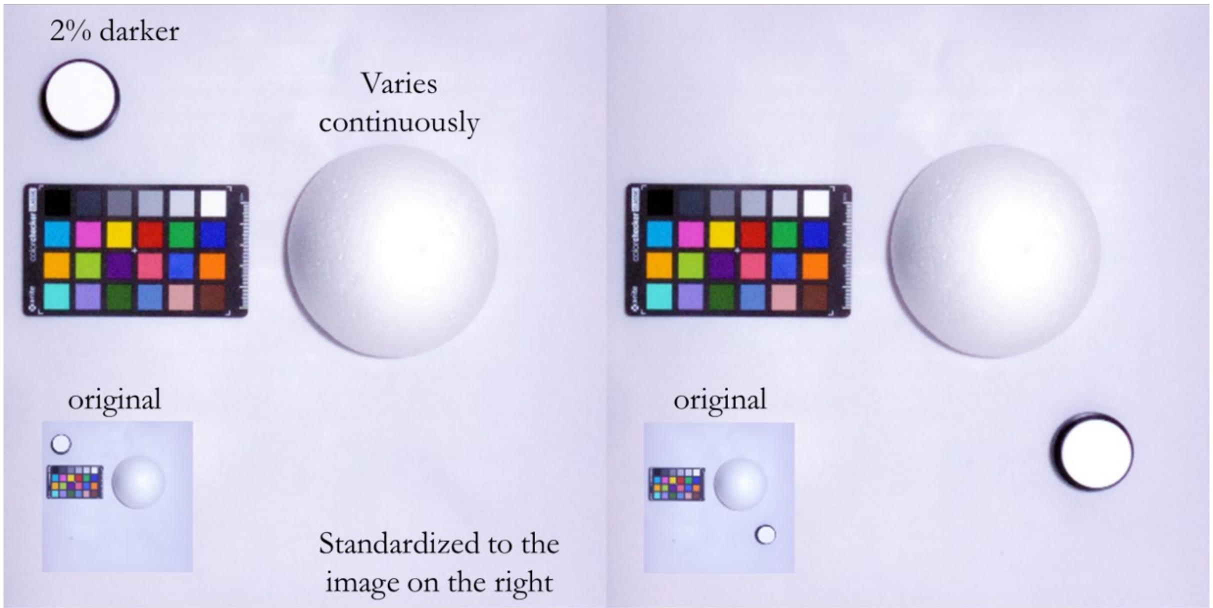

Images illustrating that standardized lighting can have a dramatic impact on measurements even of standards. Images calibrated to image on the right

Figure illustrating that the light shed by standardized bulbs disperse in a predictable fashion

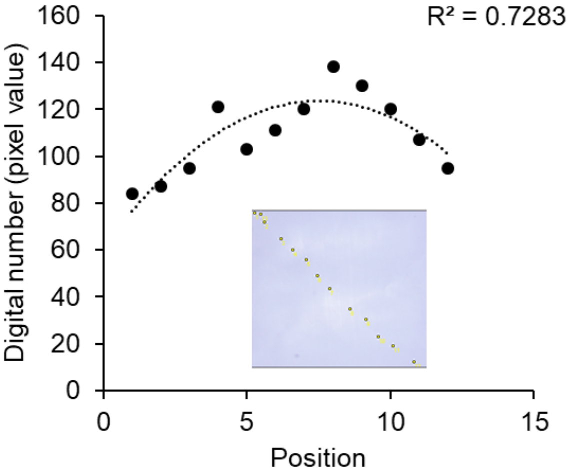

(lightest at the center). Taken on virgin PTFE sheet; however 'wrinkles' in sheet add noise.

Perhaps this isn't a 'problem' since, as you stated in the 'Hyperspectral imaging: future challenges and opportunities' section (after all, most colors are not viewed under perfectly diffuse conditions); however, even if that's the case, more details on your decisions would be useful because in the interim, myself and others may adopt your decisions until more data are available to make other decisions. How does the physical setup of your workflow remove such artifacts or allow an end user to circumvent or measure and account for these effects?

My goal would be to buy the camera, set up the lighting, image a specimen, and pass it through your workflow. To me the practical details on the configuration of the lighting are not described thoroughly enough for me to do this. Sure, I do know the supplies, but I wouldn't feel confident I would mount the lights, backing material, etc. in a way that would allow me to replicate these data.

Summary: Please provide more complete information on how you have controlled lighting in your pipeline, where the lights are mounted relative to the sample, how the light is diffused (if at all), etc., so it will be easier to implement.

Highlighting potential applications

You made a strong case for your approach on measuring the King of Holland's bird-of-paradise. The strong emphasis of the (admittedly interesting) biology of this example, seemed to preclude a broader discussion of what would be possible. I would encourage you to provide some extra direction. This would be a game changer for studying insect coloration, egg coloration, nest coloration, feather coloration (though I think you have that covered), flower coloration, and even for forensic investigations, etc. Since your technique could have a really broad appeal, I would appreciate a little extra attention to really show off a bit. Since the readership will have broad interests, but perhaps not immediately realize how they can use it. I'm not asking for you to provide data on other system, but reducing the emphasis on the case-study and increasing your discussion on scope will help.

In addition to the research areas outlined above, I think it would be nice to elaborate on the importance of dSh and dW. If this is one of the contributions of your python workflow, this could be incredibly important. There is great utility in separating the brightness from spectral shape, which you hint at on lines 63-67 of your supplement. I think that is the information appropriate for the main text, and that some of the information about these parameters from the main text *might* be supplemental. Personally, I am frustrated by the fact that most visual spaces that I use ignore brightness. We've adapted some 'phenotypic' spaces that try to get around this; however, they make assumptions about the visual system that aren't ideal for every end-user's application. This could be immensely useful to me, and I suspect others. I might highlight that this could be helpful in the main text more explicitly.

You mention fluorescence (lines 798-809) but your focus (see my point #1) is more about the mechanisms underlying the property and why it is present in one parent species but not the other parent or the hybrid. While there are interesting questions about pigments and genetics, I think this is a bit of a missed opportunity to have novel methods to quantify and separate reflected colors from their fluorescence emissions. One *could* exclude excitation bands and compare

images produced under full spectrum light, visible light (excitation^{-}), and UV light (excitation^{+}). The benefit of your approach is that you would obtain full spectra over the full body, which will help us disentangle these components of coloration (there are many reasons why this will be helpful). Some users may even feed light through a filter wheel, linear variable filter, or monochromator (or similar) to tune to specific excitation bands. Overall, I think there would be a benefit of shifting the focus from the biological interpretation here (after all there are too few specimens) and instead use this to highlight (yet another) unique and novel feature of what you are proposing.

One note on this: It may be worth using another UV torch to 'document' this. Some structures do not fluoresce at ~390nm but will at ~365nm. It's hard to find high power UV torches, but have found the $5-10 DarkBeam torch (sold on Amazon) is very effective. The last time I checked it *was* actually peaking at the reported ~365 (they have a more standard 390nm variety as well). It may be worth your investing. You may be able use a UV-monochromator and reflectance spectrometer to confirm the excitation spectrum more precisely. If you do not have enough power (likely), then I recommend placing a short pass filter in front 365nm DarkBeam torch and then photograph the specimen in the dark using a standard camera. This would isolate the emission alone (one might also subtract away the non-fluorescent blue light, by producing a second image where you cut only the UV from the 395nm torch – if e.g., you only have access to a longpass filter).

Summary: If you reduce the emphasis and focus on the King of Holland's bird-of-paradise, please consider elaborating on the other (multiple) significant benefits your pipeline offers. In an attempt to help, I've highlighted just a few in this section – there are others.

Clearer description of the pipeline itself

I would like to know more explicitly what the workflow does. A large portion of the writing seems to be a call to more widely adopt commercially available hyperspectral photography. Similarly, there is a lot of discussion about hybridization. I imagine that all of the analyses and visualization is handled by your workflow and pipeline; however, the lines were blurred a bit about what was your codes did in all of this. It would be great to really showcase what your codes enable more specifically that would be great, since only through them would this be truly helpful to the readers. I wouldn't want to change the language massively, but ideally, I would buy the hyperspectral camera, set up lighting for photography (see point 2), obtain specimens, image calibration using the Resonon software, then process using your software. At first reading (Line 156), I thought your python codes interfaced with the camera directly because it states that it includes "imaging," but then on line 265 it is clear that the images are first processed by Resonon software. My hope is that I could immediately apply the technique. Critical to that would be a clearer description of when I feed data into your custom scripts and what those scripts do, in a more explicit manner (i.e., what is Resonon software and what is a custom script). I think this could be addressed in a single sentence as the majority of what you have done was created through your python codes.

I thought that other details needed explanation, e.g., the extrapolation of the wavelength range (Lines 342), the choice of densities (SI Line 123), the calibration targets from S14 that are presumably incredibly useful for photogrammetry, etc. There are many practical questions that are left unanswered.

Summary: Please more clearly articulate your pipeline. Elements may involve the physical set up, software from Resonon, how data are collected, and then how this is fed into your python scripts. A clearer emphasis on the full process and which pieces of that pipeline are being proposed here would be helpful to highlight the novelty of the work (and help it be used)

Minor comments:

Line 160: While I do not disagree that birds are great subjects for this technique, I think that I wouldn't over-emphasize this because there are many others including beetles, plants, eggs, nests, etc. Your approach should help *all* of these, and the tone here suggests that perhaps this is more useful for birds than these other taxa/specimens, and I disagree.

Line 173: I think this extra information about taxonomic debates is unnecessary and distracting. I think you should simply refer to these as *Cicinnurus* and then state that you follow the convention outlined in 39.

Lines 174-183: While fascinating, I do not feel that lines 174-183 particularly advance your case for hyperspectral imaging. Simply stating that it is a hybrid with (apparently) intermediate features would suffice.

Lines 193-208: Again, while generally helpful, I think this could be reduced substantially. I think that the main point is that these feathers are likely colored through pigments, structure, and a combination. Your approach will let you capture the products of all of these types of colors.

Line 218: "intermediacy" sounds a bit awkward, perhaps consider rewording.

Line 278: "including noise data outside of the range of sensitivity" seems worthy of elaboration.

Line 342: I think it would be very useful to expand on how you expanded the spectrum into the 300-325nm range. This is not immediately, clear. Did you force the smoothing spline through the origin (or minimum value considering noise)? Since there is so little UV light in that region and the camera isn't sensitive to it, that would be reasonable. Also, were these extrapolations ground-truthed using standard reflectance spectroscopy?

Line 369: You compared all pixels to all other pixels? Was this for both dSh and dW? I wonder if this is actually necessary? It would generate very large datasets, but you are interested in relatively modest regions of interest – I suspect this may be overkill a pipeline limitation. What are the system requirements? A potential solution to this would be to allow the user to select ROI – which is admittedly work for a *future* paper, but I think the ideas from this line deserve expansion.

Line 371: This "attempts" and, presumably succeeds, right?

Line 376: Here you state that the data generated are amenable to statistical analysis, but aren't explicit about the analyses. You might benefit from showing these, unless they require you to have many more specimens.

Line 397: Here you raise a very interesting point; however, the loss from converting between camera-independent and animal-dependent is quite small. There is a -real- benefit to your method, but I'm pretty sure it does not lie in the 0.01% that is lost on this conversion. Instead, I would argue a far larger problem with the camera-to-animal transformations is that they make substantial assumptions on the visual systems that we map to, which are often based on samples sizes of 1. This is a limitation of both approaches though. Any minor errors from space conversions are likely to be swamped out entirely by errors introduced from misplaced assumptions. Essentially the error isn't from the conversion per se but really that multispectral photography has only a few data points (e.g., 4 color channels) where you have many more and can obtain reflectance spectra.

Line 408: This is of great important and also applies to Spectralon standards!

Line 445: I think that "from π to -π" requires more explanation. I assume these are used to describe the motion of the object to camera, but it may also refer to the color space, etc.

Line 628: I think that developing a simple developmental model probably deserves more attention and explanation. This is, independently, quite interesting and should have more than a passing mention (surely a lot of work).

Line 705-706: This is your main point and an excellent point. It would be fantastic if this could be emphasized throughout in a more complete way.

Line 735: One argument against this approach could be that we will sometimes miss meaningful color patches that we cannot see.

Line 744: now published.

https://journals.plos.org/plosbiology/article?id=10.1371/journal.pbio.3002444

Line 810: Great story, but as I see it this is less directly related to the main thrust of the paper. There is *so* much more than can be explored with these hybrids, but little of that would be necessary to provide your method and resource or convince me of its value. See point 1 above.
