# Supplementary material for: Hyperspectral imaging in animal coloration research: A user-friendly pipeline for image generation, analysis, and integration with 3D modeling
Source: PLoS Biol. 2024 Dec 3;22(12):e3002867. doi: 10.1371/journal.pbio.3002867 (PMC11614258; doi:10.1371/journal.pbio.3002867)
Supplement: Attachment — Submitted filename: Hogan_Stoddard_ResponseToReviewers.pdf [file pbio.3002867.s004.pdf]

Dear Dr Hogan,

Thank you for your continued patience while your manuscript "Hyperspectral imaging is a powerful tool for animal coloration research: A case study of a rare hybrid bird-of-paradise" was peer-reviewed at PLOS Biology as a Methods and Resources Article. Please accept my sincere apologies for the delays that you have experienced during the peer review process. Your manuscript has now been evaluated by the PLOS Biology editors, an Academic Editor with relevant expertise, and by three independent reviewers. Please note that Reviewer #2 has provided the review as a PDF document which is attached to this decision letter.

In light of the reviews, which you will find at the end of this email, we would like to invite you to revise the work to thoroughly address the reviewers' reports.

As you will see, the reviewers are generally very positive about your hyperspectral imaging pipeline and think the method will be useful for the field. However, Reviewers #2 and #3 raise some overlapping concerns, including the overall framing of the paper and noting that a greater emphasis should be placed on the methodological advance. In addition, Reviewer #3 notes that the manuscript lacks direct benchmarking to other existing methods in order to demonstrate a proven advantage. After discussions with the Academic Editor, we agree that this would be a valuable addition and we ask that direct benchmarking experiments are included in the revision. Finally, Reviewer #2 asks that additional reporting details should be included for the lighting and elaborating on other applications offered by the approach.

Given the extent of revision needed, we cannot make a decision about publication until we have seen the revised manuscript and your response to the reviewers' comments. Your revised manuscript is likely to be sent for further evaluation by all or a subset of the reviewers.

We expect to receive your revised manuscript within 3 months. Please email us (plosbiology@plos.org) if you have any questions or concerns, or would like to request an extension.

At this stage, your manuscript remains formally under active consideration at our journal; please notify us by email if you do not intend to submit a revision so that we may withdraw it.

We thank the PLOS Biology Editors, the Academic Editor, and three reviewers very much for their helpful comments and suggestions. The Editor had three major suggestions for our revision:

1. Greater emphasis on the methodological advance, with reduced emphasis on some of the current discussion on birds-of-paradise

We have reduced the emphasis on the birds-of-paradise, focusing more on the merits of the hyperspectral imaging as a methodological advance.

2. Direct benchmarking to existing methods

Reviewer 3 writes, ""e.g. How does it compare to other methods in practice (show evidence)? Would alternative conclusions be reached if this study if other (cheaper/easier) methods were used (i.e. direct comparison of the output of methods, or modelling of circumstances where high spectral resolution is required?)" We do not believe that Reviewer 3 is asking for benchmarking *per se* but is instead requesting that we be more explicit about our comparisons with other approaches. We have made it substantially clearer that we expect that hyperspectral data generation allows both prior analytical approaches (utilizing high-resolution spectral data via spectrophotometry and high-resolution spatial data via multispectral photography) to be accessible and improved by data collection using hyperspectral imagers. In that sense, our approach is not in competition with other approaches, but a consolidation of them. We have added new details to the text (see Lines 140-146, 281-292, 736-748, 79-796 and our detailed response below) to address these comments.

3. Additional details on lighting and other applications offered by the approach

We now include more details on the lighting in lines 245-263 and include a new figure in the SI (Fig S1) to illustrate the lighting set up. We have expanded on other applications, see lines 734-740, and detailed responses below.

We address the specific reviewer comments below.

Reviewer #1:

GENERAL

A splendid paper outlining a potentially transformative new approach to avian color quantification. I want to highlight, in particular, the beautiful and informative figures. The authors strike a nice balance among justifying this new approach relative to two others, describing the pipeline and potential applications, and illustrating its use with an interesting example. I have mainly minor comments throughout, see below for details.

Thank you for these generous comments.

No methods section? Can methods and results be separated? Not sure about journal format, but L233-455 read like methods and L456-678 read like results.

Thank you for the comment. We have adjusted the section titles to better match both your suggestions and typical formatting.

Figure 2: Put hybrid image between parentals, logical and aligns better with subsequent figures.

Thank you—we have made this change.

RESULTS

L242: Double-check your figure panel letters here.

We have checked and updated the references to figures in this paragraph, and we have checked for similar errors throughout.

L261: Commentary about missing those shortest 25 nm here or elsewhere? These species don't have much UV reflectance, but how could this affect species with more prominent UV patches? Might be interesting to do an analysis validating the extrapolation technique.

Note also that reviewer #3 notes that avian visual sensitivity is not high in the range 325 – 300 nm, so suggests that the issues arising may be minimal. We added the following (L351-356):

“We then extrapolated reflectance values in the wavelength range 300-325nm by applying a cubic 1D monotonic smoothing spline fit to the range 325nm -700nm (see Fig S2 for a figure contrasting ‘raw’ sampled spectra with filtered and extrapolated versions, and see Fig S3 for a figure contrasting ‘raw’ and filtered and extrapolated natural spectra). Because birds have limited sensitivity to light at wavelengths shorter than 325 nm, we anticipate that results for visual system dependent analyses will be very robust to extrapolation.”

We include in the SI a new analysis that inspects the fit of extrapolated and smoothed natural spectra with their raw counterparts, which shows that the extrapolation should not lead to spurious results <325nm.

L457: This section is somewhat redundant with some previous sections. I think you've already clearly made the case for hyperspectral imaging and how/why it's different/better than the other two methods (e.g., Table 1).

We shortened this paragraph but would like to retain it as a brief reminder of the goal of the pipeline—to generate high-quality hyperspectral data—before focusing on the more specific insights gleaned about the Birds-of-Paradise.

Figure 5: This is the only figure with embedded descriptive text, could be removed and covered in the caption.

This is a good point. After careful consideration, we feel that including some text in this figure will allow readers to understand the figure quickly. We will defer to the editors as to whether this is permissible according to journal style.

L495: I would rethink the use of the word "favors" here. "Resembles" might do.

We have changed the subtitle to "The hybrid King of Holland's bird-of-paradise has plumage colors that are generally intermediate between its parent species, but it more closely resembles one or the other parent in different patches"

L552: I think "further" should be "farther" here, as this refers to an actual and not metaphorical distance. Look for other instances of this throughout the ms if there are any.

Thank you for this catch – we have corrected this and checked other uses of 'further.'

DISCUSSION

L742: I think this section could be expanded upon to discuss the potential limitations of basing color measurements/comparisons off specimens alone (e.g., effects of age, preservation method/quality, metadata availability).

We have added the following section following your thoughtful comment (L724-733):

"For now, hyperspectral imaging for animal coloration as implemented here may be largely limited to museum specimens. However, museum specimens do not always perfectly represent the colors of live animals. For example, the physical posture for the

specimen chosen during preparation can strongly influence the colors visible on the animal surface. Further, as specimens age, their color can change or fade, especially if the specimen is exposed to sunlight [3]. Lastly, complete metadata is often unavailable for historical specimens, sometimes limiting their use for study. Hyperspectral imaging is currently primed for imaging animal specimens, but in situ animal color measurements will have to wait for advances in hardware (such as video-speed capture) to allow rapid collection of full-frame hyperspectral data with high spatial and spectral resolution and broad wavelength sensitivity."

We note that the later bird-of-paradise focused section discusses the failure of museum specimens to reflect bare-part color too.

L753: Watch for capitalization consistency in species names.

We have checked the capitalization consistency.

Reviewer #2:

Dear Drs. Hogan and Stoddard,

Thank you for the opportunity to review this very exciting Method and Resource manuscript. In this manuscript you propose the broader application of hyperspectral photography for quantifying museum data. The project uses a commercially available system and a workflow that you have designed, which should allow for your technique to be adopted broadly. Finally, you provide an ecologically and evolutionary meaningful example, by way of the King of Holland's bird-of paradise. This is a nice example not only because it is visually appealing, but because it (and its parent species) provide a nice array of colors to showcase the method. I'm very supportive of your manuscript and would be eager to use it myself!

Thus, I imagine there will be wide appeal among the PLoS Biology readership. What I'm most pleased by was how you described why such a system is useful; this technique provides users with spatially explicit color information, without compromising spectral resolution. In fact, you will see that I actually would like to see more of this. Most importantly the technique provides data that makes few assumptions. Therefore, once specimens are processed their data can be used by you, me, or any other user to ask new questions.

As you can tell, I'm very supportive of your work, so much so that I can't wait to adopt it! That being said, I do think there are areas that could be improved. I will outline these below in detail, but they broadly fall into the following categories:

1. Emphasis

2. Details on lighting

3. Highlighting potential applications

4. Clearer description of the pipeline itself

Apologies for the length of my comments for these major critiques. Hopefully these outline my points clearly. I want to avoid you needing to write lengthy responses back, so I have provided an underlined summary for each major comment. Hopefully these make it easier for you to have one response back to each major category, rather than very many responses. In addition, I have listed a few minor suggestions following these larger comments. I hope that you find my feedback useful and constructive.

Thank you,

Daniel Hanley

Dr. Hanley: Thank you very much for your comprehensive review of our work! We appreciate your detailed comments, which we have aimed to address (described below). We are excited that the method could be useful to you and others in the community.

Major comments:

Emphasis

As a Methods and Resources contribution, I would really be looking for greater emphasis on the method and less on the interesting biological contribution. I understand that it is imperative to communicate the unique contribution your method will make for the field; however, I think that you have made that case, and it could be better integrated into the description of the method itself. While, I really love the discussion of the hybrid bird-of-paradise my overall suggestion would be to devote less space in outlining their biology (background, descriptions, implications, etc.) and more on how your method allowed you to record data that will address these questions. In this case, I believe you have imaged 1 specimen of each species which seems sufficient to describe the method but less sufficient to fully address the biology. I feel it would be better if the emphasis could shift more to hyperspectral imagery and more specifically the workflow you designed.

Naturally, you will want to follow the recommendations of the editors on this point; however, it was something that was emphasized to our lab when we recently wrote for this outlet, and hinted at in the review invitation, “While we value the inclusion of novel biological insight in our 'Methods and Resources' articles, this is not a condition for publication of this article type in PLOS Biology.” I loved your biological insight, but think it could be reduced and the resource description could be expanded.

Summary: Please consider reducing the emphasis on the biological interpretation and refocus your emphasis on the contributions of your new pipeline and the practical considerations on how one might use that pipeline.

Thank you for the careful explanation of your concerns, several of which were shared by Reviewer #3.

In line with these suggestions, we have made several cuts to the introduction to better focus the reader on the techniques and methods:

177-183

187-196

202-208

216-225

524-532

643-649 & 665-682: and we have modified the corresponding figure (7) to remove the developmental model of tail rachis curvature, in line with this comment and more specific comments from Reviewer #3. Details of the rachis model can now be found in the SI.

764-772

786-793

800-807

As for increased emphasis on the methods, we have added more details as explained under the relevant comments below.

Details on lighting

Your method is tantalizing; however, there are many practical considerations that I am still unsure how I would tackle if I were to adopt this today. The largest of these has to

do with controlling light while measuring your specimens. You did discuss what light source you were using, and importantly even described the importance of considering the effect of directional light sources on measurements and interpretation. However, I found details lacking in this regard and frankly wouldn't be able to adopt this workflow without knowing. For example, was your xenon arc lamp in free air in a custom fixture? If so, which type. Bulbs like this (see bulb in image) are typically in a housing (see housing in image), which provide strong directional lighting and provide adapters for various ways to cast the light. If the light was cast through optics what type were used. I imagine you may have reflected the light off of a surface and had that cast light on your subject, or used UV-VIS diffusers (e.g., from Edmund Optics).

Thank you for this note: we have added more details on the equipment used to direct the light in the following section (L245-256):

"We illuminated specimens in these images using a UV-rich and spectrally broadband light source (450W Xenon ozone free arc lamp: Newport Corp., Irvine, CA, powered with a Newport OPS-A1000 power-supply). This light source was housed in a rectangular enclosure (Newport 66921 arc lamp housing), the output of which was first filtered for infrared light by passing through a water filter filled with deionized water (Newport 6123NS liquid filter) and then directed vertically downward using a UV-reflective turning mirror (beam turning mirror: Newport 66246, beam turning mirror housing: Newport 66246). The light, enclosure, filter and turning mirror were placed on a small rolling table, so that the turning mirror could be placed as close to the lens of the hyperspectral imager as possible. This resulted in bright directional light, from approximately the same angle as the imager's receptive field, cast over the entirety of the push-broom field of view of the hyperspectral imager (see Fig S1). One limitation of this lighting set-up is that objects closer to the light source will appear artificially lighter. Therefore, improving the control and directionality of incident light is a goal for future work (see Discussion)."

We hope that this section, in addition to a new figure in the supplemental information (Fig S1), helps to shed some light on the equipment and set-up used.

Furthermore, I would be eager to know more about where these lights were placed relative to your samples. I hate to be picky here, but these are the questions I would want answers to when I adopt your technique. I also think they are vitally important. When photographing specimens, I aim to diffuse the light as much as is possible, not because I think this replicates nature, but because our specimens are measured with reference to standards. Light from these bulbs has a distinct and predictable power spectrum that means some areas of the image will receive more light. Therefore, the

same 99% Spectralon standard will not necessarily have the same value in the top right corner as it will in the bottom left (there's a 2% shift in the image below). Naturally, if this is the case, it will impact your accuracy of the specimens as well. To account for this, I've seen some authors use full field calibrations but don't think that's necessary in your case. This tends to occur with indoor photography more than natural light, which is preferred. Below are a few illustrative images taken of standards under a free-air bulb (~1.25 m above the specimens). You also mention the impact of shape, which invariably impacts colors even if consistently colored (e.g., the Styrofoam hemisphere), and even slightly angling your Spectralon can impact it's color by up to 20% (from flat to angled at ~45 degrees).

Images illustrating that standardized lighting can have a dramatic impact on measurements even of standards. Images calibrated to image on the right

Figure illustrating that the light shed by standardized bulbs disperse in a predictable fashion (lightest at the center). Taken on virgin PTFE sheet; however 'wrinkles' in sheet add noise.

Perhaps this isn't a 'problem' since, as you stated in the 'Hyperspectral imaging: future challenges and opportunities' section (after all, most colors are not viewed under perfectly diffuse conditions); however, even if that's the case, more details on your decisions would be useful because in the interim, myself and others may adopt your decisions until more data are available to make other decisions. How does the physical setup of your workflow remove such artifacts or allow an end user to circumvent or measure and account for these effects?

My goal would be to buy the camera, set up the lighting, image a specimen, and pass it through your workflow. To me the practical details on the configuration of the lighting are not described thoroughly enough for me to do this. Sure, I do know the supplies, but I wouldn't feel confident I would mount the lights, backing material, etc. in a way that would allow me to replicate these data.

Summary: Please provide more complete information on how you have controlled lighting in your pipeline, where the lights are mounted relative to the sample, how the light is diffused (if at all), etc., so it will be easier to implement.

Thank you for the thoughts (and figures!) on this topic. In addition to the modifications discussed above (including additional figure in supplement, Fig S1) we would like to note that we do something similar to a 'full field calibration' here, as stated on line 256;

“Each image was calibrated spatially (i.e., we corrected for non-uniformity in lighting across the field of view of the imager) using SpectrononPro.”

This stage of processing uses a board of PTFE that encompasses the entire field of view of the imager to take a ‘bright’ image with which to calibrate all other data being recorded. Note that this is much easier for a push-broom imager than for a full frame camera (as in your figures) because only a single line needs to be lit and filled by the bright constant object. Division of the data by the values measured from this bright and constant object allows us to account for differences in the light field as best we can (as well as any differences in sensitivity between pixels in the sensor). To make that clearer, we add the following section to the above (L258):

“This process involves collecting a ‘bright image’ of a rectangular block of white PTFE that encompasses the entire field of view of the imager, which is used to account for differences in brightness across the field of view due to directional lighting”

Of course, this doesn’t fully address the scientific concern which is that directional light cast on a 3D object cannot be fully accounted for by any calibration (bar perhaps a 3D facsimile of the 3D object made of Spectralon!). Thank you for noting our section in the discussion on this topic. We feel that solving this problem is work for the future, but we hope the additions to the manuscript would give the reader the tools to more confidently set up the lighting as we did.

Highlighting potential applications

You made a strong case for your approach on measuring the King of Holland’s bird-of-paradise. The strong emphasis of the (admittedly interesting) biology of this example, seemed to preclude a broader discussion of what would be possible. I would encourage you to provide some extra direction. This would be a game changer for studying insect coloration, egg coloration, nest coloration, feather coloration (though I think you have that covered), flower coloration, and even for forensic investigations, etc. Since your technique could have a really broad appeal, I would appreciate a little extra attention to really show off a bit. Since the readership will have broad interests, but perhaps not immediately realize how they can use it. I’m not asking for you to provide data on other system, but reducing the emphasis on the case-study and increasing your discussion on scope will help.

In addition to the research areas outlined above, I think it would be nice to elaborate on the importance of dSh and dW. If this is one of the contributions of your python

workflow, this could be incredibly important. There is great utility in separating the brightness from spectral shape, which you hint at on lines 63-67 of your supplement. I think that is the information appropriate for the main text, and that some of the information about these parameters from the main text might be supplemental. Personally, I am frustrated by the fact that most visual spaces that I use ignore brightness. We've adapted some 'phenotypic' spaces that try to get around this; however, they make assumptions about the visual system that aren't ideal for every end-user's application. This could be immensely useful to me, and I suspect others. I might highlight that this could be helpful in the main text more explicitly.

Following the suggestions of Reviewer #3, we have shifted the focus of the paper away from the KLPD approach somewhat. We agree with Reviewer 3 that PCA (and in our case, not subtracting the mean value from each spectrum, so explicitly retaining brightness information) is more conventional. In practice, with our dataset, the results are equivalent. We now explain the KLPD approach in the SI—and we have added a few sentences about the usefulness of dSH and dW there. We share your frustration with the common emphasis on chromaticity over brightness!

You mention fluorescence (lines 798-809) but your focus (see my point #1) is more about the mechanisms underlying the property and why it is present in one parent species but not the other parent or the hybrid. While there are interesting questions about pigments and genetics, I think this is a bit of a missed opportunity to have novel methods to quantify and separate reflected colors from their fluorescence emissions. One could exclude excitation bands and compare images produced under full spectrum light, visible light (excitation-), and UV light (excitation+). The benefit of your approach is that you would obtain full spectra over the full body, which will help us disentangle these components of coloration (there are many reasons why this will be helpful). Some users may even feed light through a filter wheel, linear variable filter, or monochromator (or similar) to tune to specific excitation bands. Overall, I think there would be a benefit of shifting the focus from the biological interpretation here (after all there are too few specimens) and instead use this to highlight (yet another) unique and novel feature of what you are proposing.

This is a very interesting suggestion, though the work is outside of the scope of this paper. We have added a sentence in the fluorescence section (L779):

"Further characterization of the fluorescence, including specifics of the excitation and emission wavelengths, might also be studied using a hyperspectral imager in concert with spectral filters and/or a monochromator."

One note on this: It may be worth using another UV torch to 'document' this. Some structures do not fluoresce at ~390nm but will at ~365nm. It's hard to find high power UV torches, but have found the $5-10 DarkBeam torch (sold on Amazon) is very effective. The last time I checked it was actually peaking at the reported ~365 (they have a more standard 390nm variety as well). It may be worth your investing. You may be able use a UV-monochromator and reflectance spectrometer to confirm the excitation spectrum more precisely. If you do not have enough power (likely), then I recommend placing a short pass filter in front 365nm DarkBeam torch and then photograph the specimen in the dark using a standard camera. This would isolate the emission alone (one might also subtract away the non-fluorescent blue light, by producing a second image where you cut only the UV from the 395nm torch – if e.g., you only have access to a longpass filter).

Thank you for the tips!

Summary: If you reduce the emphasis and focus on the King of Holland's bird-of-paradise, please consider elaborating on the other (multiple) significant benefits your pipeline offers. In an attempt to help, I've highlighted just a few in this section – there are others.

Thank you for this constructive suggestion! We agree that fluorescence imaging is a great avenue for future use of the hyperspectral imager in addition to the ideas noted above. We add a section L734-740 under the '*Hyperspectral imaging: future challenges and opportunities*' section:

"Although our study focused on plumage colors, hyperspectral imaging holds great promise for the study of animal color in diverse invertebrate and vertebrate taxa, including butterflies [27,33], beetles [29], spiders [28], and cephalopods [31], to name a few. Hyperspectral imaging is likely to yield new insights about the evolution of color and pattern in bird eggs, bird nests, fruit and flowers—and could reveal rich details about animal color in the contexts of urbanization and climate change. Similar insights might be gleaned from spectrophotometry and multispectral imaging, too—but only hyperspectral imaging provides high-resolution spectral and spatial data simultaneously."

Clearer description of the pipeline itself

I would like to know more explicitly what the workflow does. A large portion of the writing seems to be a call to more widely adopt commercially available hyperspectral photography. Similarly, there is a lot of discussion about hybridization. I imagine that all

of the analyses and visualization is handled by your workflow and pipeline; however, the lines were blurred a bit about what was your codes did in all of this. It would be great to really showcase what your codes enable more specifically that would be great, since only through them would this be truly helpful to the readers. I wouldn't want to change the language massively, but ideally, I would buy the hyperspectral camera, set up lighting for photography (see point 2), obtain specimens, image calibration using the Resonon software, then process using your software. At first reading (Line 156), I thought your python codes interfaced with the camera directly because it states that it includes "imaging," but then on line 265 it is clear that the images are first processed by Resonon software. My hope is that I could immediately apply the technique. Critical to that would be a clearer description of when I feed data into your custom scripts and what those scripts do, in a more explicit manner (i.e., what is Resonon software and what is a custom script). I think this could be addressed in a single sentence as the majority of what you have done was created through your python codes.

I thought that other details needed explanation, e.g., the extrapolation of the wavelength range (Lines 342), the choice of densities (SI Line 123), the calibration targets from S14 that are presumably incredibly useful for photogrammetry, etc. There are many practical questions that are left unanswered.

Summary: Please more clearly articulate your pipeline. Elements may involve the physical set up, software from Resonon, how data are collected, and then how this is fed into your python scripts. A clearer emphasis on the full process and which pieces of that pipeline are being proposed here would be helpful to highlight the novelty of the work (and help it be used)

Thank you for these comments. We have modified the initial section describing the pipeline to aid in clarity. To note, we consider that the code we present is just a part of the analysis pipeline – and that imaging (using Resonon's SpectrononPro software) is included. L219-225:

"The pipeline consists of three steps: i) imaging, ii) sampling, and iii) low-dimensional embedding (see Fig 3). Imaging involves acquiring images from the hyperspectral camera (Fig 3A) using SpectrononPro software (Resonon Inc., MT, USA). The subsequent sampling and embedding steps are performed using custom python code (available at XXX). Sampling involves selecting relevant colors from the hyperspectral images for analysis (Fig 3B-D). Low-dimensional embedding involves generating informative transformations of the spectral data for interpretation and analysis (Fig 3E-F)."

We also moved a section relating to extrapolation of wavelengths from the "imaging" section to the "sampling" section, and we added a section to "imaging" as follows: L242-244 "Data collection and initial spatial calibration were conducted using SpectrononPro software (Resonon Inc.)" in order to more clearly delineate what was undertaken in Resonon's SpectrononPro software, and what was undertaken in Python.

We modified the section L275-292 to read:

"These data-cubes were saved in a standard band-interleaved-by-line (BIL) format, along with a header file which contains metadata. The data-cubes produced by SpectrononPro software averaged 3.2 gigabytes in size. Hyperspectral data-cubes can be analyzed in a number of software packages/environments including SpectrononPro software, MATLAB (MathWorks, Natick, MA), Python (Python Software Foundation, Wilmington, DE. Available at www.python.org), and ENVI (NV5 Geospatial Solutions, Inc., Broomfield, CO). In the current study, we used custom code to extract and further analyze sets of spectra from the hyperspectral images in Python (v3.7.4, see all subsequent steps of the pipeline), but one of the key advantages of hyperspectral imaging is that the data are amenable to both image-based and spectra-based color analyses. For example, it is possible to apply visual models to entire hyperspectral images to generate cone stimulation images which can then be analyzed using current packages, including MICA/QCPA for ImageJ [2,66]. We include in our code an example of this. Alternatively, users may be interested only in one or a small number of wavelength bands of interest. These may be easily extracted and analyzed as individual images. Yet another approach might be to extract only a few spectra for further color analysis using the R package pavo [67]. We include in our code examples of this approach (and see section 'Some King of Holland's bird-of-paradise color patches that are spectrally intermediate would be indistinguishable from those of its parent species when modeled with avian color vision using only one spectrum per patch')."

We hope that this makes it clearer that data are generated in SpectrononPro, and all analysis is undertaken using Python code.

Minor comments:

Line 160: While I do not disagree that birds are great subjects for this technique, I think that I wouldn't over-emphasize this because there are many others including beetles, plants, eggs, nests, etc. Your approach should help all of these, and the tone here suggests that perhaps this is more useful for birds than these other taxa/specimens, and I disagree.

Thank you for this suggestion, we agree! As noted above, we added a section in the Discussion that we hope better highlights the utility of hyperspectral imaging across a broad range of animal coloration areas (L734-738):

"Although our study focused on plumage colors, hyperspectral imaging holds great promise for the study of animal color in diverse invertebrate and vertebrate taxa, including butterflies [27,33], beetles [29], spiders [28], and cephalopods [31], to name a few. Hyperspectral imaging is likely to yield new insights about the evolution of color and pattern in bird eggs, bird nests, fruit and flowers—and could reveal rich details about animal color in the contexts of urbanization and climate change"

Line 173: I think this extra information about taxonomic debates is unnecessary and distracting. I think you should simply refer to these as Cicinnurus and then state that you follow the convention outlined in 39.

We have made this change. This sentence now now reads:

"*C. magnificus*; following the convention used in [39]"

Lines 174-183: While fascinating, I do not feel that lines 174-183 particularly advance your case for hyperspectral imaging. Simply stating that it is a hybrid with (apparently) intermediate features would suffice.

Thank you. We have significantly reduced this section. See our response to your summary on 'Emphasis' for a list of cuts.

Lines 193-208: Again, while generally helpful, I think this could be reduced substantially. I think that the main point is that these feathers are likely colored through pigments, structure, and a combination. Your approach will let you capture the products of all of these types of colors.

Thank you. We have significantly reduced this section. See our response to your summary on 'Emphasis' for a list of cuts.

Line 218: "intermediacy" sounds a bit awkward, perhaps consider rewording.

This section was removed in response to earlier suggestions.

Line 278: "including noise data outside of the range of sensitivity" seems worthy of elaboration.

Thank you for pointing this out; we hope the following addition is clearer (L273):

“Visual inspection of the data indicates that bands below 325nm contain only noise, which means that in our configuration, this system is sensitive from 325nm to 700nm, encompassing almost the complete range of avian visual sensitivity (300nm – 700nm).”

Line 342: I think it would be very useful to expand on how you expanded the spectrum into the300-325nm range. This is not immediately, clear. Did you force the smoothing spline through the origin (or minimum value considering noise)? Since there is so little UV light in that region and the camera isn’t sensitive to it, that would be reasonable. Also, were these extrapolations groundtruthed using standard reflectance spectroscopy?

We have expanded this section somewhat to add nuance, and to make it clear that we do not believe that extrapolation is always necessary, or that we are strongly advocating for a particular method (L343-356).

“We then interpolated spectra to 1nm bands (from 325nm to 700nm) and smoothed the spectra by application of a Savitzky-Golay filter (window 45nm, polynomial order 2). It is possible that interpolating spectra to a spectral resolution of greater than 1nm would reduce computational complexity of the analyses, potentially at little cost. We note that the values of these smoothing parameters were chosen here to aid in stable extrapolation of reflectance values below 325nm (where small amounts of noise around 325nm produce large differences in the resulting extrapolation), but that the result is relatively extreme smoothing (see Fig 1, 3, S2, S3). Future research should determine levels of smoothing appropriate for varied applications. We then extrapolated reflectance values in the wavelength range 300-325nm by applying a cubic 1D monotonic smoothing spline fit to the range 325nm -700nm (see Fig S2 for a figure contrasting ‘raw’ sampled spectra with filtered and extrapolated versions, and see Fig S3 for a figure contrasting ‘raw’ and filtered and extrapolated natural spectra). Because birds have limited sensitivity to light at wavelengths shorter than 325 nm, we anticipate that results for visual system dependent analyses will be very robust to extrapolation.”

We also add to the SI a figure (Fig S3) that shows 90 randomly selected spectra from a separate spectrophotometric dataset (natural spectra of plumage and plants) overlaid with versions of the spectra processed as the ones in the main paper were. This should help the reader to see that extrapolation usually generates reasonable results, but sometimes fails. Also relevant here is an additional SI figure (fig S2) with two panels, one showing ‘raw’ sampled spectra, and the other showing the same data after filtering and extrapolation. We hope that the reader now has enough information.

Line 369: You compared all pixels to all other pixels? Was this for both dSh and dW? I wonder if this is actually necessary? It would generate very large datasets, but you are

interested in relatively modest regions of interest – I suspect this may be overkill a pipeline limitation. What are the system requirements? A potential solution to this would be to allow the user to select ROI – which is admittedly work for a future paper, but I think the ideas from this line deserve expansion.

We are here explicitly talking about the 'sampled reflectance spectra', following the section on "Extracting sample reflectance spectra from hyperspectral images" which states that:

"Sampling from the hyperspectral images allowed us to i) control the computational complexity of the analysis, and ii) reduce redundancy due to spatial autocorrelation (i.e., pixels tend to share the color properties of neighboring pixels). We first manually generated for each image a set of regions-of-interest indicating the locations of pixels within select body parts/patches: whole (the entire body), breast (the pectoral shield as per [68]), belly/vent (all of the ventral side of the bird, below the pectoral shield excluding the legs), shoulder, and back (including wings, see Fig S11-15 for details of the placement of these patches). To sample colors from each of these patches, we defined a uniform grid of sampling locations in each image, the spacing of which was chosen to approximately equalize the number of sampling locations in images of each patch within a specimen, and across all specimens (Fig 3B)."

So we are in fact generating distances among only a subset of the total reflectance spectra found in each hyperspectral image; as per your suggestion.

In fact, on the suggestion of reviewer 3, we concentrate more on the PCA approach than our original KLPD approach in the revised manuscript, which obviates the pairwise comparisons.

Line 371: This "attempts" and, presumably succeeds, right?

Interesting point. The embedding of N-dimensional pairwise distances into lower dimensional space will not be perfect; these algorithms attempt to minimize stress/error in the final distances in relation to the initial distances. We think 'attempts' is appropriate here, though we understand the linguistic awkwardness.

Line 376: Here you state that the data generated are amenable to statistical analysis, but aren't explicit about the analyses. You might benefit from showing these, unless they require you to have many more specimens.

As you rightly note we don't have many specimens, and our aim is more a characterization and exploration of the colors of the birds than a statistical analysis. We

cite Cuthill et al 1999 at the end of the line in question, in the hope that readers interested in developing statistical analyses with a PCA version of the exploration we show here might start looking there. We add the following text for additional places to start (L384):

"Our goal here is to illustrate and explore the spectra rather than to conduct statistical analyses, but PCA coordinates are amenable to statistical approaches: for instance, one might look for statistical separation in the distributions of points of each species (e.g., using MANOVA)."

Cuthill IC, Bennett ATD, Partridge JC, Maier EJ. Plumage reflectance and the objective assessment of avian sexual dichromatism. The American Naturalist. 1999 Feb;153(2):183–200.

Line 397: Here you raise a very interesting point; however, the loss from converting between camera-independent and animal-dependent is quite small. There is a -real-benefit to your method, but I'm pretty sure it does not lie in the 0.01% that is lost on this conversion. Instead, I would argue a far larger problem with the camera-to-animal transformations is that they make substantial assumptions on the visual systems that we map to, which are often based on samples sizes of 1. This is a limitation of both approaches though. Any minor errors from space conversions are likely to be swamped out entirely by errors introduced from misplaced assumptions. Essentially the error isn't from the conversion per se but really that multispectral photography has only a few data points (e.g., 4 color channels) where you have many more and can obtain reflectance spectra.

We agree with this, but we also suggest that the sensitivity of the camera and lens are also substantially assumed in many multispectral workflows, since characterizing cameras is difficult and expensive. It's true though that only some workflows depend on exact characterization of the camera and lens (i.e., those that do not use color-checkers or similar). We think that there is a distinct benefit in eliminating all the technical assumptions, as we state – but you're right that ultimate accuracy also depends on the visual model that we are using. The benefits of hyperspectral imagery in terms of spectral resolution are discussed in the introduction and discussion (for example, L76-91), and a new SI figure (Fig S4) hopefully illustrates to readers your point that the low spectral resolution of multispectral imaging might introduce errors for some natural spectra.

Line 408: This is of great important and also applies to Spectralon standards!

Very true! See our responses to comment above.

Line 445: I think that “from π to -π” requires more explanation. I assume these are used to describe the motion of the object to camera, but it may also refer to the color space, etc.

We clarify this in the caption for Figure 4:

“These images indicate the surface normal of the specimen relative to the camera in color from π to -π. The color indicates the horizontal component (roll, C) and vertical component (pitch, D) of the specimens’ surface normal relative to the camera, with values close to 0 signifying surfaces nearly parallel to the camera, and more extreme values indicating extreme angles relative to the camera.”

We add the clarifying lines:

“For roll (C), π indicates that the surface normal of the specimen is facing to the left of the camera and -π indicates a normal facing to the right. For pitch (D), π indicates a surface normal pointing up relative to the camera, and -π indicates a downward angle”

Line 628: I think that developing a simple developmental model probably deserves more attention and explanation. This is, independently, quite interesting and should have more than a passing mention (surely a lot of work).

This section was removed to better focus on the methodology employed, rather than on birds-of-paradise–and in response to a comment by Reviewer #3. We’re encouraged that you find it interesting, since we couldn’t find much information on how the rachis becomes curved. We may explore this idea further in future work, but for now the details can be found in a new section in the SI.

Line 705-706: This is your main point and an excellent point. It would be fantastic if this could be emphasized throughout in a more complete way.

Thank you. We hope that the revised text, with greater focus on methods and less focus on the specifics of the bird-of-paradise example has emphasized this point more clearly.

Line 735: One argument against this approach could be that we will sometimes miss meaningful color patches that we cannot see.

We fully agree, and we have changed the final line to better represent what we were getting at (L714):

“It seems likely that soon some combination of these efforts will result in a robust means by which to identify discrete patches in an objective and automated way, free of human biases.”

Line 744: now published.

https://journals.plos.org/plosbiology/article?id=10.1371/journal.pbio.3002444

Thank you, this reference is now updated.

Line 810: Great story, but as I see it this is less directly related to the main thrust of the paper. There is so much more than can be explored with these hybrids, but little of that would be necessary to provide your method and resource or convince me of its value. See point 1 above.

This section has been removed, in line with editor and reviewer suggestions that we focus more on the method and less on the specific BOP example.

Reviewer #3: This paper showcases a pipeline for hyperspectral imaging of museum specimens, and uses it to investigate the provenance of a putative hybrid bird of paradise.

Overall I really like the methods, and wholeheartedly welcome the release of open-source tools for the community. The manuscript is nicely written, though I feel it could be shortened in parts. Hyperspectral imaging techniques will be of increasing interest to a wide audience, and birds-of-paradise are a charismatic group to investigate.

I make a large number of minor points below, but none should be difficult to deal with, it mostly reflects the fact that (I think) I understand the methods quite well.

I have just two main concerns (again, easy to address, but might entail some work). One trying to work out the rationale for this paper, the other technical.

Dr. Troscianko: Thank you very much for your thorough and thoughtful review. We appreciate your constructive comments and suggestions!

Rationale: The core hypothesis being tested is not entirely convincing as-is. e.g. deciding whether a species of apparently intermediate coloration/physiology between two other species really represents a hybrid would ideally need a full phylogenetic comparison (measure lots of species with this technique, including other known hybrids), or at least some meaningful out-group, control or reference. As such, there's no clear alternate hypothesis. For example, the statement that the (alleged) hybrid is 'strikingly

intermediate' has no baseline for comparison. I don't know how much the authors want to sell this finding as important though, as I feel the real value of the paper lies in its methodology (which is great), and this hypothesis is just used as a demo. In any case it's shortcomings are worth discussing. However, if the authors do want to sell the methodology there are some important things I'd like to see to convince readers that this method is worth using themselves.

We agree with you (and reviewer #2 and the editors) that the focus should be more methods-focused and less about birds-of-paradise, and that the paper therefore could be shortened. With that aim, we have cut several sections to better focus the reader on the techniques and methods:

177-183

187-196

202-208

216-225

524-532

643-649 & 665-682: and we have modified the corresponding figure (7) to remove the developmental model of tail rachis curvature (now found in the SI)

764-772

786-793

800-807

e.g. How does it compare to other methods in practice (show evidence)? Would alternative conclusions be reached if this study if other (cheaper/easier) methods were used (i.e. direct comparison of the output of methods, or modelling of circumstances where high spectral resolution is required?)

This is a fair point. In theory, if you very densely sampled a specimen with a spectrophotometer, you could replicate the spectral analyses. Likewise, if you took images with a multispectral camera, you could replicate a spatial analysis. We have tried to make it clearer throughout the manuscript that hyperspectral imaging is useful because it can achieve, with one instrument, what *together* a spec and multispectral camera can. We also now clarify that the analyses conducted post-imaging are just a suggestion/demonstration of the *types* of analysis that can be done. They are not *in lieu*

of or superior to analyses that could be performed in pavo or MICA/QCPA. We have added the following text (Lines 284-292):

"For example, it is possible to apply visual models to entire hyperspectral images to generate cone stimulation images which can then be analyzed using current packages, including MICA/QCPA for ImageJ [2,66]. We include in our code an example of this. Alternatively, users may be interested only in one or a small number of wavelength bands of interest. These may be easily extracted and analyzed as individual images. Yet another approach might be to extract only a few spectra for further color analysis using the R package pavo [67]. We include in our code examples of this approach (and see section 'Some King of Holland's bird-of-paradise color patches that are spectrally intermediate would be indistinguishable from those of its parent species when modeled with avian color vision using only one spectrum per patch')."

See also a section that reads (L745):

"Future software should ideally be freely available, highly computationally efficient (and amenable to cloud computing), and inter-operable with existing tools for multispectral image analysis (such as MICA/QCPA [2,5]) and spectral analysis (such as the R package pavo [67])."

And the following (L790-794):

"Using the example of the birds-of-paradise, we provide a guide for capturing and analyzing hyperspectral data, which we hope other researchers will adopt and update. We also provide example code in which hyperspectral data can be easily incorporated into existing spectra- and image-based color analysis toolkits (pavo: [67], MICA/QCPA: [2,5])"

In addition, we now include a figure to the SI which we hope will show some readers the kinds of data loss they might expect when evaluating natural spectra with only a limited number of color channels, as in multispectral imaging (Fig S4).

Processing methods: (L342) I'd like more information on the rationale for the sampling and smoothing applied here. And importantly I'd like to see some raw data before any smoothing is applied. 45 nm is a surprisingly spectrally wide window, and 1nm a surprisingly high spectral resolution. This stage will be computationally inefficient as it's artificially increasing the spectral resolution from that of the native hardware. This will also cause all following calculations to be equally inefficient for no apparent gain in accuracy. Up-sampling might give false impressions of spectral resolution. Why not just downsample the entire cube to e.g. a 10nm resolution that wouldn't need smoothing,

and would give pretty similar effects for less computational overheads? Or even more elegant, just work with the spectral range you're given by the camera and adjust the bin-width in the calculations as necessary (as these will presumably be non-linear).

Thank you for this thoughtful comment. We considered this carefully and have added two figures to the supplemental material (see Fig S2,3). We also now clarify in the main text (L343-356):

"We then interpolated spectra to 1nm bands (from 325nm to 700nm) and smoothed the spectra by application of a Savitzky-Golay filter (window 45nm, polynomial order 2). It is possible that interpolating spectra to a spectral resolution of greater than 1nm would reduce computational complexity of the analyses, potentially at little cost. We note that the values of these smoothing parameters were chosen here to aid in stable extrapolation of reflectance values below 325nm (where small amounts of noise around 325nm produce large differences in the resulting extrapolation), but that the result is relatively extreme smoothing (see Fig 1, 3, S2, S3). Future research should determine levels of smoothing appropriate for varied applications. We then extrapolated reflectance values in the wavelength range 300-325nm by applying a cubic 1D monotonic smoothing spline fit to the range 325nm -700nm (see Fig S2 for a figure contrasting 'raw' sampled spectra with filtered and extrapolated versions, and see Fig S3 for a figure contrasting 'raw' and filtered and extrapolated natural spectra). Because birds have limited sensitivity to light at wavelengths shorter than 325 nm, we anticipate that results for visual system dependent analyses will be very robust to extrapolation."

We interpolate from ~2.5nm non-linear bands to 1nm linear bands because we consider this to be a relatively conventional way of dealing with spectral data (for instance it is used in R package pavo by default). You are right that computational efficiency would be improved with lower resolution calculations, maybe at little cost. That said, we don't feel like the analysis is particularly time-intensive - all sampling and all calculations (KLPD, PCA, umap embeddings for each, and human and avian color representations, etc) for one patch (for example the 'whole' patch) across all 24 hyperspectral images of all 6 specimens takes 5 ½ minutes. A lot of that time is taken up by the colour-science's package slow generation of human-visible colors.

We have now added the line (L345):

"It is possible that interpolating spectra to a spectral resolution of less than 1nm would reduce computational complexity of the analyses, potentially at little cost."

We have also added a little analysis of the flower and plumage spectral dataset in which we overplot the data interpolated to 1nm, with a 90nm interpolation (to generate 5 channels, somewhat like a multi-spec – although without integration across a wide band of wavelengths). We plot the spectra with the greatest maximum distance between the 1nm and 90nm data, so that readers can get a grasp on what maximum loss is likely on some natural colors.

We also added a SI figure with two panels, directly contrasting the same 'raw' sampled spectra and the same but extrapolated and filtered. Our goal in this figure is to 1) show what we're doing to our data and 2) to indicate that we're not particularly advocating this specific approach but instead pointing users to do evaluate their own research needs.

Related question: L336 It strikes me that the processing pipeline would be far more efficient if each pixel were first converted to avian cone-catch values (or non-system-specific bins), and then the rest of the analysis done simply on conventional images. This doesn't strike me as particularly computationally tricky or slow. Even a single processor should take less than a minute to churn through a 5MPx200 channel cube with efficient code (python might not be great for this heavy lifting though). I haven't seen the code, but would like to have a look,

This is an interesting point. We collect samples for each patch from *multiple* images, rather than from only one image, and for the visual system-independent spaces, the space itself is defined by the variability across the samples (from multiple images) themselves (excluding samples from outside of the ROIs on each image), so we are not sure it's true that this exact approach would be either simple or computationally more efficient as an image processing task. For instance, running a PCA on a whole hyperspectral image and taking samples would not result in the same space as taking samples and running PCA on them.

At any rate, we feel these kinds of changes are outside of the scope of the paper. We do recognize that for visual system-dependent whole image analyses, mica/QCPA already has a strong framework that there's no need for us to replicate here. We've added to our code the means by which a user might readily integrate a hyperspectral image with a visual model and save it into a multi-page 32-bit float TIFF image compatible with forward image processing with mica/QCPA.

Minor points:

It's worth pointing out that high spectral resolution is not always required. Natural reflectance spectra and solar illumination rarely have sharp changes across a 5-10nm range. This means we can infer the way colours will look to a given animal under given (natural) lighting with a fairly high degree of confidence. The reflectance profiles shown in fig.1 for example, would behave very predictably at lower spectral resolution sampling. Where this fails is when either the light source is non broadband (important for my current work in artificial light pollution), or when reflectance profiles are particularly rare (e.g. sometimes achieved in structural coloration).

It’s true that, especially for visual models, high resolution is often not required. It’s also true that most natural spectra are pretty smooth. However, whether and how much data are lost with reduced spectral resolution are pretty variable. The high spectral resolution of this imager would capture all that information. We’ve demonstrated this with a new figure in the SI, where we overlay separately collected natural spectra (plumage and plants) with a subsampled spectrum (of 5 channels, evenly spaced for simplicity), and then found the examples with the greatest difference between the resulting spectra. We hope that will help the reader to understand what kinds of spectra are problematic when downsampling, and what nuances in the spectra might be missed. This of course is mostly relevant to visual system-independent analyses, when the spectra are not subsequently analyzed with an animal’s photoreceptor sensitivity curves. There are of course many non-signaling contexts in which having a complete reflectance spectrum is desirable (i.e., for predicting the structural or pigmentary color production mechanism).

L20 I'd query whether hyperspectral data are computationally costly. It depends on the spectral, spatial, and temporal resolution, but the hardware has typically been the limiting factor here. I also query the affordability below.

You’re right. We’ve rephrased this to “Fortunately, costs are decreasing and hyperspectral imagers are improving, particularly in their sensitivity to wavelengths (including ultraviolet) visible to diverse animal species”

Table 1. I'd like a further row of "temporal resolution" added. This highlights the issue with using multispectral and hyperspectral cameras and moving targets. e.g. some systems can work instantaneously, or capture video, but others require slower scanning/repeat photos taking. The camera used here requires scanning, so cannot take instantaneous images. It's perhaps worth referencing Vasas et al's recent dichroic camera PLoS Biol paper here as an example of low-spectral, but high spatial and temporal resolution (you cite the pre-print, but can cite the full thing now).

We now include an additional row about temporal resolution to the table, as you suggest. Since some hyperspectral imagers are full-frame, and hyperspectral video exists, we indicate that hyperspectral imaging temporal resolution is ‘typically low’; we make the same designation for multispectral photography, since most UV-Vis applications are only suitable for stationary scenes.

As you note, we already have a section in the discussion on this topic, but we have expanded this section (L716-733):

“At present, hyperspectral imaging typically has relatively poor temporal resolution (Table 1). Hyperspectral imaging of live animals moving in the wild (at least with high spectral and spatial resolution, and broad wavelength sensitivity) is not yet practical because images take minutes to generate. To some extent, this problem is also true of multispectral photography, although a study recently introduced the use of a beam splitting mirror to photograph a scene using ultraviolet and visible light sensitive cameras simultaneously (typically at framerates up to 30 per second), allowing the collection of multi-spectral video [84]. Both hyperspectral imaging and multispectral photography may benefit from better integration of imaging with 3D modelling and animation, where dynamic animal motion may be introduced and investigated in silico ([23,85]). For now, hyperspectral imaging for animal coloration as implemented here may be largely limited to museum specimens. However, museum specimens do not always perfectly represent the colors of live animals. For example, the physical posture for the specimen chosen during preparation can strongly influence the colors visible on the animal surface. Further, as specimens age, their color can change or fade, especially if the specimen is exposed to sunlight [3]. Lastly, complete metadata is often unavailable for historical specimens, sometimes limiting their use for study. Hyperspectral imaging is currently primed for imaging animal specimens, but in situ animal color measurements will have to wait for advances in hardware (such as video-speed capture) to allow rapid collection of full-frame hyperspectral data with high spatial and spectral resolution and broad wavelength sensitivity.”

L77 multispectral imaging doesn't always use one camera spectral channel for one receptor channel (though often does). Worth just rephrasing slightly. e.g. people often model achromatic and dichromatic vision using RGB cameras.

We have rephrased this as suggested.

L91 Multispectral and hyperspectral data are both able to generate reflectance spectra. It's simply a matter of resolution and range. Indeed, conventional spectrometry can't

handle the full UV-vis-IR-thermalIR ranges required to understand all the effects discussed here.

You’re right, but the preceding lines read (L77):

“Although reflectance spectra (high spectral resolution) and multispectral images (high spatial resolution) are both amenable to visual modeling and can be used to address a range of questions about how animals perceive color signals (reviewed in [13,14]), for …”

We feel that the parenthetical here should let the reader know that we’re talking about the benefits of high spectral resolution spectra in this section.

L104 'brightness' is not the right word here. The system will be measuring radiance (specifically energy of light in a known solid angle or a known spectral range).

Quite right, corrected.

L141 Could you say how much the system used here cost? This would be valuable for readers. Include the broadband stable lighting setup used (these can be super expensive too), plus the ~$10,000 UV-VIS lens. It'll all add up quite a bit. Presumably $20-30k? Which dents the angle that this is affordable.

We state that the systems are “becoming increasingly affordable”, not that they are now affordable but you’re quite right that an estimate of the cost might be appropriate here. We have now included in the discussion an estimate of the total cost of all equipment used in this study (L740-743);

“Hyperspectral imaging systems remain expensive; Resonon is currently selling its UV-sensitive imaging system for about $55,000 USD, and extra lights and lenses can add to the bottom line. However, we expect that costs will continue to decrease in the future.”

254 Personally I'd like to see other colour spaces added. e.g. the RNL Chromaticity colour space is much easier to use, interpret, and analyse with reference to discriminablility (though I'd caution that almost all colour spaces are pretty flawed, so it's not worth getting too fussy).

Thank you for this idea. We’ve implemented this in our code and added to the SI a plot of the RNL chromaticity using equations from Renoult et al (2017). We also agree that color spaces have pros and cons—and the RNL chromaticity space makes some assumptions about suprathreshold discrimination that have not yet been validated.

L247 Are you getting at measuring gloss and iridescence here? i.e. goniometry? If so, maybe be explicit. You bring this up later at least, but generally there's scope of more concise writing.

This sentence just serves to prime the reader that an additional optional step involving 3D models will be discussed at the end of the ‘methods section, and later in the discussion. On consideration, we think that it is a useful signpost.

L264 This spatial correction is interesting, presumably normalising each pixel to account for non-diffuse lighting. But this highlights real problems for scanning/imaging anything that's not flat. Things nearer the lights than the 2D white calibration tile will appear artificially lighter. So for 3D subjects illuminated from non-diffuse, close light sources care needs to be taken for accurate reflectance estimates.

We agree. We have now added more information about the simple approach to lighting that we used (and include in the SI a diagram of the lighting set up Fig S1), and we tell the reader explicitly that in the future this could be improved (L245-256):

“We illuminated specimens in these images using a UV-rich and spectrally broadband light source (450W Xenon ozone free arc lamp: Newport Corp., Irvine, CA, powered with a Newport OPS-A1000 power-supply). This light source was housed in a rectangular enclosure (Newport 66921 arc lamp housing), the output of which was first filtered for infrared light by passing through a water filter filled with deionized water (Newport 6123NS liquid filter) and then directed vertically downward using a UV-reflective turning mirror (beam turning mirror: Newport 66246, beam turning mirror housing: Newport 66246). The light, enclosure, filter and turning mirror were placed on a small rolling table, so that the turning mirror could be placed as close to the lens of the hyperspectral imager as possible. This resulted in bright directional light, from approximately the same angle as the imager’s receptive field, cast over the entirety of the push-broom field of view of the hyperspectral imager (see Fig S1). One limitation of this lighting set-up is that objects closer to the light source will appear artificially lighter. Therefore, improving the control and directionality of incident light is a goal for future work (see Discussion).”

L262 birds have very low sensitivity to UV below 325nm, and the sun doesn't give out much energy at these wavelengths either. I'm just saying this in case anyone worries that part of the range is 'missing' - it's not likely to be an issue as far as I can tell.

We added a small note: (L354)

“Because birds have limited sensitivity to light at wavelengths shorter than 325nm, we anticipate that results for visual system dependent analyses will be very robust to extrapolation.”

L288 Really happy to see use of Python (great to keep code open source). But is it all in python? As an interpreted language it's not always very quick for image processing.

Yes, it’s all in Python, although where possible we rely on the numpy library which implements basic mathematical functions in C. Elsewhere, we make use of the SpyFile format from Spectral Python to avoid loading whole HSIs into memory, instead reading only what is necessary from data.

L355 I disagree here - cameras are frequently used to compare spectral reflectance. e.g. NDVI methods, and enormous numbers of methods and studies. I get what the authors are hinting at, but the only real difference with the system used here is its spectral resolution, not the ability of the system to measure spectral reflectance across a given spectral range. Indeed, even common light meters (not cameras) often work by comparing a 'full-spectrum' to an infrared measurement to determine brightness in the visible range.

We’ve updated this sentence to reflect this concern (additions underlined, L367):

“Because this approach requires embedding high-resolution reflectance spectra, it would not be possible to produce an identical color space using multispectral photography”

L376 Why not just focus on the PCA-based method then? I like the PCA method, and it's used a fair bit in the field (as you say, it's easy to understand and apply stats etc...). Could make the paper more concise, and help the readers.

We carefully considered this suggestion, and we agree that there are merits to PCA. We have now updated the manuscript so that the PCA approach is discussed in the main text, and the KLPD approach is discussed in the SI. We have added the section (L373-386):

“To embed the spectra in a low dimensional system-independent color space, we begin by applying principal components analysis (PCA, implemented using scikit-learn [69]) to our sampled spectra (one PCA per plumage patch). We elected not to subtract the mean reflectance from each spectrum (see [7]) because we were not more scientifically motivated to investigate the influence of spectral shape than brightness/reflectance. The calculated PCA coordinates (20 dimensions) are not easily interpretable since any two-

dimensional plot (for instance axis 1 versus axis 2) may not fully capture the distances between the embedded colors. To reduce this dimensionality, we used Uniform Manifold Approximation and Projection, (UMAP: [68], see below and SI) in order to embed spectra in a lower dimensional space that attempts to maintain distances between PCA coordinates. Spectra that fall close together in this space will tend to be those with low distances between PCA coordinates, and those far apart will tend to have high distances. The result is a single, easily interpretable 2D plot of the distances among sampled spectra. Our goal here is to illustrate and explore the spectra rather than to conduct statistical analyses, but PCA coordinates are amenable to statistical approaches: for instance, one might look for statistical separation in the distributions of points of each species (e.g., using MANOVA)."

We have also updated the main text figures to represent the new focus on PCA > KLPD, and updated the relevant SI figures.

L408 But you are only measuring each point once or twice without actively controlling the angles, so unlike goniometry you can't quite work out iridescence with this method (without more work).

We have added the qualifier 'at the patch level' to this sentence. We hope that this, in addition to the section in the Discussion, makes it clear that we're looking at the angle-dependence of the patch as a whole rather than an individual point.

L535 How sure can we be that these differences aren't due to noise of some sort? Also, all spectral recordings will differ, but how can we say different any two are without some point of reference or threshold?

We have softened this sentence by adding 'likely'. We think it's true that our data show that patches have more than one spectral shape in them, and that one or a few spectra would 'likely' not 'adequately describe the variety of spectral shapes present'. This is agnostic as to whether the spectral shapes are meaningfully different, and in any case what is or is not meaningfully different is going to depend on the context of the study and system. It could be true that all of those spectra collapse into one location in a chromatic space, but that does not mean that variation in spectral shape means nothing to a researcher interested in pigment composition, for instance, or in brightness or gloss. See lines 612-61:9

"While a visual system-independent approach uncovered some differences between colors, for instance in breast coloration, visual models indicate that not all of these differences are perceptually relevant (when assessed using a single spectrum for each

patch, but see Fig S17). This analysis highlights the importance of tailoring the approach (visual system-independent vs. visual system-dependent) to the scientific question at hand. If we were interested in the evolution of pigmentation in the context of signaling behavior, a visual system-independent approach may produce irrelevant or even misleading results. In contrast, if we were interested in the physical or optical properties of pigmentation irrespective of visual system, the situation would be reversed."

L590 If you're converting to RNL values why use the tetrahedral colour space at all? Just convert to RNL XYZ chromaticity, then you have your hue axes just like the tetrahedral space, but now Euclidean distance is equal to RNL delta-S values (i.e. perceptually uniform-ish). Hue and discriminability can be compared in one go.

Thank you for the idea to implement RNL color space; we have done so. However, we feel that this section is still informative as it is because it also allows us to discuss luminance (dL) distances at the same time. We point the reader toward the new SI figure using the RNL space showing that each patch contains probably many discriminable colors (even within species), and we in the section title that this original analysis is using only one spectrum (average) per specimen.

L595 What visual system (spectral sensitivity curves, Weber fraction and cone ratios do you use here?)

You're quite right that we omitted those details – thank you for catching this (L391-397).

This section now reads: "Here, to estimate how a bird-of-paradise might perceive the plumage colors of the King, Magnificent and hybrid King of Holland's bird-of-paradise, we embedded each of our sampled reflectance spectra in a tetrahedral model of avian vision [64,65] assuming ideal illuminant and background. Birds-of-paradise are presumed to have violet-sensitive (VS) visual systems [70], so we used visual pigment sensitivities for an average violet-sensitive bird (extracted from the R package pavo [67]), as well cone ratio values of (1:1:1:2) reported for Rock dove (Columba livia) [71] and chromatic and achromatic Weber fractions of 0.1."

L628 I like this method for modelling feather growth. Though maybe you could have just fitted a bezier spline? It might be worth mentioning why developmentally-inspired methods are used rather than just absolute modelling here, but this approach is not used for spectral reflectance curves (even though the discussion goes over many developmentally relevant colour creation mechanisms)? Playing devil's advocate, this is a lot of work and effort to say something that is obvious to any viewer of the feathers (the

intermediate one is intermediate). I really like the methods, but their use doesn't seem enormously well justified here - persuade me I'm wrong.

In accordance with editor and other reviewer comments, in order to focus more on the methods, we've moved the section on the tail growth model to the SI for interested readers.

L703 As above, I'm not convinced there are meaningful differences shown between spectra of the same patch. What exactly would 'meaningful' be given everything in the world has some degree of difference?

We have rephrased as follows (L675): "Further, we found that, in general, patches are not homogenous: summarizing plumage using single or average color spectra may obscure substantial differences within patches".

L798 Fluorescence is a pig! Relating to my above point, artificial light sources aren't able to simulate how something looks if strong fluorescence is involved as it violates the illuminant/reflectance equations. Few people realise this, so it's perhaps worth pointing out to readers. Your use of xenon lighting was fairly good in this regard, and moderately likely to replicate natural viewing (although the UV component will be far higher than sunlight). Most other light sources will create more errors in inferred reflectance.

Thank you for raising this important point. We've added the section (L774):

"We note that strong fluorescence can complicate the measurement of animal color; if the light source does not approximate natural lighting, the light produced by fluorescence can introduce errors in inferred reflectance (pers. comm. Jolyon Troscianko)"

The discussion gets very speculative regarding bird of paradise crosses, and to be honest I don't find it particularly useful if the thrust of this paper is methods. The bird of paradise based work here doesn't shed light on many of the issues touched on in the discussion.

We have now reduced the focus on the birds-of-paradise, as described above.
