# Supplementary material for: Hyperspectral imaging in animal coloration research: A user-friendly pipeline for image generation, analysis, and integration with 3D modeling
Source: PLoS Biol. 2024 Dec 3;22(12):e3002867. doi: 10.1371/journal.pbio.3002867 (PMC11614258; doi:10.1371/journal.pbio.3002867)
Supplement: Attachment — Submitted filename: PBiology-D-23-03297_r2 comments.pdf [file pbio.3002867.s005.pdf]

Dear Dr. Hogan and Stoddard,

Great work on your revision. I appreciate your detailed responses to all three reviews, and feel that your revised manuscript is greatly improved. You present a really compelling and exciting case for adopting hyperspectral images. Previously, I felt that your work focused too much on a specific (interesting) case of hybridization, and too little on the method you were proposing. This is no longer the case. In my opinion the current version strikes a very nice balance between presenting a new method, while using the interesting case of hybridization as a compelling example.

I no longer have any major critiques; however, I have provided a few comments that might help. Some are purely formatting (e.g., legible figures and axes) with which the journal can surely assist, others appear to be with your color depictions (e.g., the generated standard RGB image in Fig. 1 and 4 appear saturated and they shouldn't), and finally I have included a handful of minor suggestions on wording or flow.

Overall, this is a really great project and I wish you all the best with it!

Best wishes,

Daniel Hanley

Comments:

Line 130/Fig 1: It would be helpful to note that this is the dorsal view. Your image in B is oddly saturated, which I think is an error because none of the raw values shown in A or C are saturated. Just as an example, merging the color channels from images at 430nm, 550nm, and 610nm from A produces an image closer to my expectation and closer to the paintings of the birds in Fig 2 (images to the right). I applied a 0.5 gamma correction on the rightmost image to adjust the brightness. In both, the texture of the feathers is clearly visible and the colors appear closer to what we expect, whereas the image in Fig 1B (pasted below, and Fig 4) lacks detail due to its oversaturation. While simple to address, I think this is fairly important.

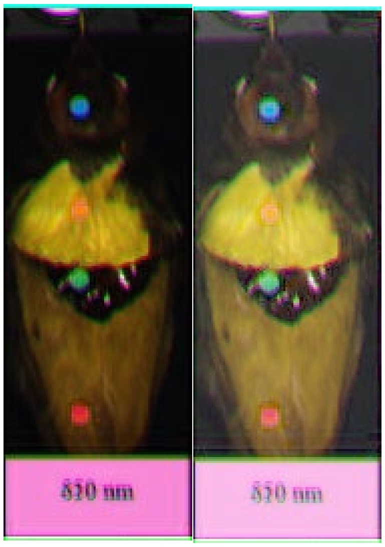

I'm not sure why the image became oversaturated, but here are a few pointers: please be sure that the max RGB value was set to 1.0 (or 255) rather than the max for the reflectance spectra recovered from (425nm, 550nm, 600nm), be sure your input values do not exceed the RGB maximum, ensure the images you used were relative to the white standard (i.e., not in counts), and double check any scaling that you may have applied. The fact that I could recolorize these from the low-res slices provided in Fig 1A, suggests that something has gone wrong with Fig 1B. The rainbow border around Fig 1B could probably be simplified unless it represents something.

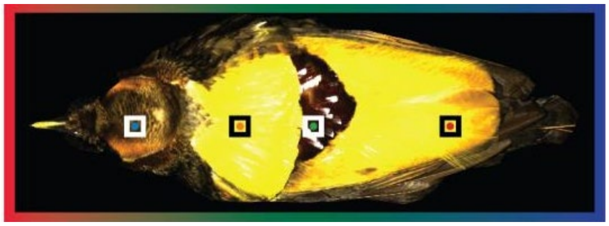

Also, for future reference, for single hyperspectral images you would have greater success in converting your pixel-level spectra into sRGB values directly.

Table 1. While I appreciate that adding temporal resolution makes your table more comprehensive, I'm not entirely sure writing low temporal resolution for all the columns is fair or necessary. In their typical application, temporal resolution for these methods seems simply 'not applicable.' However, special use-cases can capture color information quickly. For example, depending on your configuration, spectroscopy can be used to measure reflectance on the order of milliseconds (or perhaps even microseconds) to measure luminescent properties such as phosphorescence or other time-resolved spectroscopy (faster when the spectrometer and light are properly triggered). We built a newer multispectral video camera system that should be able to capture even faster motion, e.g., hummingbird displays. Ultimately, the temporal resolution depends on the configuration and can vary quite a bit. Multispectral videography may be better served as a column, but while I would love to see it represented here, it does seem to fall a little outside of the typical use cases that are relevant to your discussion. Perhaps the low temporal resolution for these methods can be more effectively highlighted within the caption?

Figure 3. Would it please be possible to make the font all equal size? Axes in Fig 3A iii, C, and D are especially hard. On my end the "H" label in a iii shows up as "1l" instead. I know that this is a little challenging when making composite images; however, many of the axes are too small (even when considering that your final resolution will be higher). You should be able to choose a font size that will end up being the same size given the final figure size and the relative size of each panel.

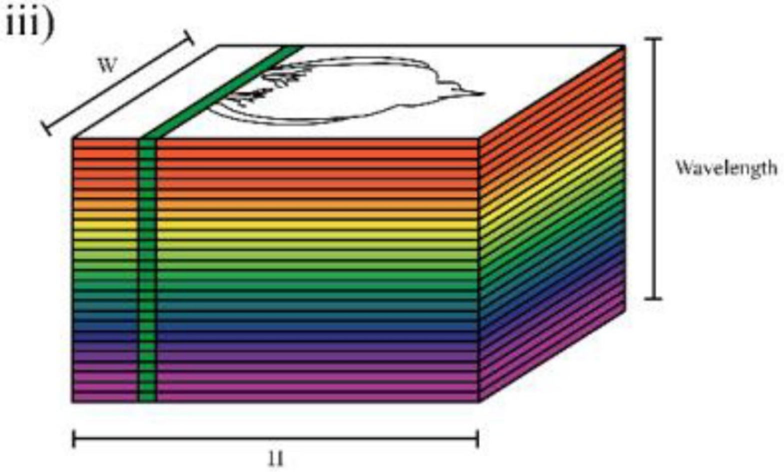

Line 373: suggested reword in brackets "… but this came at the cost of not having a uniform sampling density (samples per cm2, for instance) [, which] may be more important for other research questions."

Line 485: I would recommend removing “spatially” before 2d in this sentence.

Line 507: Please be cautious. See my comment on Fig. 1. Fig 3 seems to have reasonable RGB values, likely generated from image-generated spectra (however, is D devoid of brightness information, while C has brightness and color information?). In any case, be cautious here as there appeared to be an issue with one RGB image.

Fig 4. Like in Fig. 1 this appears saturated, and I’m 99% sure this isn’t correct. That is, none of your raw data are saturated and you should be able to produce a fine image with a very high degree of color fidelity.

Line 544: Of [each] specimen? Didn’t you do this with more than one specimen, even if much of the focus is on one specimen? Also, I’m not sure that “Thus, hyperspectral imaging is a single tool that simultaneously produces data with high spectral and high spatial resolution” follows directly from your prior sentence. Perhaps instead, “Thus, [we illustrate the utility of] hyperspectral imaging [as] a [] tool [to] simultaneously [produce] data with high spectral and high spatial resolution” Or similar

Fig. 5. Lovely image! Are the spectral peaks shown in K expected? I cannot really read to y-axes, but assume these seem reasonable to you in comparison with spectroscopy of a similar measurement geometry.

Line 591: Given the striking peak height for the hybrid, I think that it would be important to highlight that you are talking about the λ_{max} value for these curves. This should avoid unnecessary confusion. A vertical line at the λ_{max} for the hybrid could also help the reader see that the hybrid falls intermediate to the two parent species.

Lines 671: With respect to statements such as “more saturated and slightly redder in hue than the hybrid” here and throughout, should be supported by test statistics. Even though you have only a handful of specimens, you have a large population of data points from both specimens (per patch) and you have all the metrics needed (e.g., distance from the achromatic point may be a metric of saturation). This goes for other similar statements (e.g., 679-700, etc.). Unless I’m missing something, it seems reasonable to determine whether certain color metrics are, or are not, greater.

Line 886: It would be important to note that this figure of $55,000 is the base fee, and a full setup may be out reach for many researchers for many years.

Line 897: I found this switch back to the bird-of-paradise a little abrupt. I’m not 100% sure how to best address that, but perhaps some subtle shuffling of sections or connective phrases could help. That said, overall, the tone of the manuscript is striking a much better balance between the new method and the biological significance (in my opinion)

Lines 946-960: I would be a little cautious here because even if a light source matches natural lighting, spectroscopic measurements (and those from imaging) would capture radiance, rather than reflectance per se (inferred or otherwise). You could get reflectance by using a monochromator, thus separating out the reflectance from the emission spectra, as is typical in the large Perkin spectrometers used in many chemistry classrooms. Importantly, many (but not all)

museum specimens were treated with chemicals like arsenic and borax, which are known to fluoresce. One potential simple solution would be to suggest replicating your work on fresh, untreated, specimens to confirm and characterize the fluorescence (for an example, please see Travouillon et al. 2023 https://doi.org/10.1098/rsos.230325).
