# Supplementary material for: Hyperspectral imaging in animal coloration research: A user-friendly pipeline for image generation, analysis, and integration with 3D modeling
Source: PLoS Biol. 2024 Dec 3;22(12):e3002867. doi: 10.1371/journal.pbio.3002867 (PMC11614258; doi:10.1371/journal.pbio.3002867)
Supplement: Attachment — Submitted filename: Hogan_Stoddard_ResponseToReviewers2.pdf [file pbio.3002867.s006.pdf]

We routinely suggest changes to titles to ensure maximum accessibility for a broad, non-specialist readership, and to ensure they reflect the contents of the paper. In this case, we would suggest a minor edit to the title, as follows. Please ensure you change both the manuscript file and the online submission system, as they need to match for final acceptance:

“A user-friendly pipeline for the generation and analysis of hyperspectral images for animal coloration research”

Thank you for this suggestion. We agree that since the focus of the paper has shifted from the bird-of-paradise example to focus on the pipeline, a change in title is warranted. We prefer to change the title to:

“Hyperspectral imaging in animal coloration research: a user-friendly pipeline for image generation, analysis and integration with 3D modeling”

(B) You may be aware of the PLOS Data Policy, which requires that all data be made available without restriction: http://journals.plos.org/plosbiology/s/data-availability. For more information, please also see this editorial: http://dx.doi.org/10.1371/journal.pbio.1001797

Note that we do not require all raw data. Rather, we ask that all individual quantitative observations that underlie the data summarized in the figures and results of your paper be made available in one of the following forms:

-Supplementary files (e.g., excel). Please ensure that all data files are uploaded as 'Supporting Information' and are invariably referred to (in the manuscript, figure legends, and the Description field when uploading your files) using the following format verbatim: S1 Data, S2 Data, etc. Multiple panels of a single or even several figures can be included as multiple sheets in one excel file that is saved using exactly the following convention: S1_Data.xlsx (using an underscore).

-Deposition in a publicly available repository. Please also provide the accession code or a reviewer link so that we may view your data before publication.

Regardless of the method selected, please ensure that you provide the individual numerical values that underlie the summary data displayed in the following figure panels as they are essential for readers to assess your analysis and to reproduce it:

Figure 1C, 4F, 5A-N, 6A-J, 7B-D, S3, S4, S5A-N, S6A-N, S7, S8, S9, S16, S17, S21

NOTE: the numerical data provided should include all replicates AND the way in which the plotted mean and errors were derived (it should not present only the mean/average values).

We have elected to prepare all raw data in the Dryad depository, and we include all code necessary to replicate data figures.

We have additionally prepared a subfolder (Figure_dataframes.zip) in the Dryad which contains separate .csv files with the data underlying the data figures as requested.

(C) I tried to search for the hyperspectral image data deposited in the Dryad database using the DOI provided in the Data Availability Statement, but could not find it. I would be grateful if you could please make sure that the DOI is correct or to make the data publicly available at this stage.

We apologize for that oversight. Here is a reviewer link to the Dryad submission:

https://datadryad.org/stash/share/0CY5dUQDiJvo-weAc3bH4sGp5sVuYZwvTD2G1LS6zRA

Since we have changed the title of the manuscript, we are hesitant to make fully public this dataset until we can be sure that the metadata (title, abstract) match the final manuscript.

(D) Please also ensure that each of the relevant figure legends in your manuscript include information on *WHERE THE UNDERLYING DATA CAN BE FOUND*, and ensure your supplemental data file/s has a legend.

All raw data can be found on the Dryad repository, and all code to generate figures can be found on the GitHub (though see response to (F)). We've added a subfolder to the Dryad repository with each relevant figure's underlying data (Figures_dataframes.zip). We've added the following sentence to all relevant captions:

"The data underlying this figure can be found at https://doi.org/10.5061/dryad.j0zpc86nf"

We have updated the list of SI captions (at very end of MS file).

(E) Please ensure that your Data Statement in the submission system accurately describes where your data can be found and is in final format, as it will be published as written there.

We have checked and updated this statement; the DOI is correct but can only be accessed from the link provided in response to (C) until it is made public. We have archived a copy of the Github code on Zenodo (as in response to (F), https://doi.org/10.5281/zenodo.13749562).

(F) Please note that we cannot accept sole deposition of code in GitHub, as this could be changed after publication. However, you can archive this version of your publicly available GitHub code to Zenodo. Once you do this, it will generate a DOI number, which you will need to provide in the Data Accessibility Statement (you are welcome to also provide the GitHub access information). See the process for doing this here: https://docs.github.com/en/repositories/archiving-a-github-repository/referencing-and-citing-content

We've archived a release of the Github repository on Zenodo for referencing.

See: https://doi.org/10.5281/zenodo.13749562

As you address these items, please take this last chance to review your reference list to ensure that it is complete and correct. If you have cited papers that have been retracted, please include the rationale for doing so in the manuscript text, or remove these references and replace them with relevant current references. Any changes to the reference list should be mentioned in the cover letter that accompanies your revised manuscript.

We made minor edits to the formatting of some references, and we consolidated all journal references to NLM Title Abbreviations. One reference was updated from a preprint to a published article (33, Penacchio O, Halpin CG, Cuthill IC, Lovell PG, Wheelwright M, Skelhorn J, et al. A computational neuroscience framework for quantifying warning signals. Methods in Ecology and Evolution. 2024;15(1):103–16.)

We expect to receive your revised manuscript within three weeks.

To submit your revision, please go to https://www.editorialmanager.com/pbiology/ and log in as an Author. Click the link labelled 'Submissions Needing Revision' to find your submission record. Your revised submission must include the following:
- a cover letter that should detail your responses to any editorial requests, if applicable, and whether changes have been made to the reference list
- a Response to Reviewers file that provides a detailed response to the reviewers' comments (if applicable, if not applicable please do not delete your existing 'Response to Reviewers' file.)
- a track-changes file indicating any changes that you have made to the manuscript.

NOTE: If Supporting Information files are included with your article, note that these are not copyedited and will be published as they are submitted. Please ensure that these files are legible and of high quality (at least 300 dpi) in an easily accessible file format. For this reason, please be aware that any references listed in an SI file will not be indexed. For more information, see our Supporting Information guidelines:
https://journals.plos.org/plosbiology/s/supporting-information

Published Peer Review History
Please note that you may have the opportunity to make the peer review history publicly available. The record will include editor decision letters (with reviews) and your responses to reviewer comments. If eligible, we will contact you to opt in or out. Please see here for more details:
https://blogs.plos.org/plos/2019/05/plos-journals-now-open-for-published-peer-review/

We would be happy to publish the peer reviews for this publication.

Press
Should you, your institution's press office or the journal office choose to press release your paper, please ensure you have opted out of Early Article Posting on the submission form. We ask that you notify us as soon as possible if you or your institution is planning to press release the article.

Protocols deposition
To enhance the reproducibility of your results, we recommend that if applicable you deposit your laboratory protocols in protocols.io, where a protocol can be assigned its own identifier (DOI) such that it can be cited independently in the future. Additionally, PLOS ONE offers an option for publishing peer-reviewed Lab Protocol articles, which describe protocols hosted on protocols.io. Read more information on sharing protocols at https://plos.org/protocols?utm_medium=editorial-email&utm_source=authorletters&utm_campaign=protocols

---

Reviewer remarks:

Reviewer #1: Well I feel like a dolt for giving such a mild-mannered and brief review compared to the two incredibly detailed critiques you received from the other two reviewers! Thankfully

your ms made it onto the desks of two of the foremost experts in this field (plus me!). Having read through their reviews, your responses, (and the few minor responses warranted to my comments), and your revised ms, it's clear that this version has been greatly improved. In retrospect, I agree with them that - for this type of paper - the emphasis should have been more on the technique (both in terms of greater technical detail and greater discussion of applicability) than the BoP example. Your revision accomplishes this change of focus nicely. I commend the authors on a thoughtful and nuanced revision.

We appreciate your kind comments!

Reviewer #2 (Daniel Hanley, signs review):

Dear Dr. Hogan and Stoddard,

Great work on your revision. I appreciate your detailed responses to all three reviews, and feel that your revised manuscript is greatly improved. You present a really compelling and exciting case for adopting hyperspectral images. Previously, I felt that your work focused too much on a specific (interesting) case of hybridization, and too little on the method you were proposing. This is no longer the case. In my opinion the current version strikes a very nice balance between presenting a new method, while using the interesting case of hybridization as a compelling example.

I no longer have any major critiques; however, I have provided a few comments that might help. Some are purely formatting (e.g., legible figures and axes) with which the journal can surely assist, others appear to be with your color depictions (e.g., the generated standard RGB image in Fig. 1 and 4 appear saturated and they shouldn't), and finally I have included a handful of minor suggestions on wording or flow.

Overall, this is a really great project and I wish you all the best with it!

Dr. Hanley: Thank you very much for your detailed review and helpful comments!

Best wishes,

Daniel Hanley

Comments:

Line 130/Fig 1: It would be helpful to note that this is the dorsal view. Your image in B is oddly saturated, which I think is an error because none of the raw values shown in A or C are saturated. Just as an example, merging the color channels from images at 430nm, 550nm, and 610nm from A produces an image closer to my expectation and closer to the paintings of the birds in Fig 2 (images to the right). I applied a 0.5 gamma correction on the rightmost image to adjust the brightness. In both, the texture of the feathers is clearly visible and the colors appear closer to what we expect, whereas the image in Fig 1B (pasted below, and Fig 4) lacks detail due to its oversaturation. While simple to address, I think this is fairly important.

I'm not sure why the image became oversaturated, but here are a few pointers: please be sure that the max RGB value was set

to 1.0 (or 255) rather than the max for the reflectance spectra recovered from (425nm, 550nm, 600nm), be sure your input values do not exceed the RGB maximum, ensure the images you used were relative to the white standard (i.e., not in counts), and double check any scaling that you may have applied. The fact that I could recolorize these from the low-res slices provided in

Fig 1A, suggests that something has gone wrong with Fig 1B. The rainbow border around Fig 1B could probably be simplified unless it represents something.

Also, for future reference, for single hyperspectral images you would have greater success in converting your pixel-level spectra into sRGB values directly.

Thank you for your attention to detail here. We've noted that we're plotting the dorsal view for the magnificent bird-of-paradise in the caption for Figure 1 (as well as noting that it's a lateral view for the Gouldian finch in Fig 1D). We've checked and corrected the saturation issue with Fig 1B – this was due to our selection of a normalization factor (vmax). This setting was used to set the white point of the resulting RGB image to less than 100% reflectance. That's because using 100% reflectance as the max results in a very underexposed image, in which details are hard to see. We've moved this white point to a higher value, reducing apparent saturation problems here.

For all colors elsewhere, we do indeed convert from spectra to sRGB rather than simply combining 3 slices/bands – however for this figure we want to keep things quite simple for the reader – they can see the kinds of slices in question so we're just combing them. We have removed the rainbow pattered box around Fig 1 B.

Table 1. While I appreciate that adding temporal resolution makes your table more comprehensive, I'm not entirely sure writing low temporal resolution for all the columns is fair or necessary. In their typical application, temporal resolution for these methods seems simply 'not applicable.' However, special use-cases can capture color information quickly. For example, depending on your configuration, spectroscopy can be used to measure reflectance on the order of milliseconds (or perhaps even microseconds) to measure luminescent properties such as phosphorescence or other time-resolved spectroscopy (faster when the spectrometer and light are properly triggered). We built a newer multispectral video camera system that should be able to capture even faster motion, e.g., hummingbird displays. Ultimately, the temporal resolution depends on the configuration and can vary quite a bit. Multispectral videography may be better served as a column, but while I would love to see it represented here, it does seem to fall a little outside of the typical use cases that are relevant to your discussion. Perhaps the low temporal resolution for these methods can be more effectively highlighted within the caption?

We've considered this comment carefully (along with the one by Dr. Troscianko below), and we now have removed temporal resolution from Table 1. What we were trying to get at was essentially the speed of capture of color information, but as you note this is so dependent on use-case that it's probably misleading to even try to give one value for each method. For instance, one spec reading would be <1s, but in animal coloration we're often taking multiple replicate readings per patch, and measuring multiple patches. Equally, as Dr. Troscianko notes one can consider a simple RGB camera a multispectral imager, in which case one image <1s, but in some configurations (external filters) one image can be >30s. And again, if you're capturing a whole animal, many images may be required. The same really is true for hyperspectral images, some are configured as ours is (pushbroom) so that one image = some minutes, but others are full-frame imagers capable of video. So we agree that no simple entry here would be fair, and it's probably not necessary – we already devote a fair amount of time describing spectrophotometry and multispectral imaging in the introduction, and have a section on the limitations of the multi- and hyperspectral imaging in capturing moving animals in the discussion. As you suggest, we've attempted to cover these issues in the caption for the table:

"In terms of temporal resolution, we note that the amount of time to sample an individual animal is highly variable both within and among techniques. For spectrophotometry, individual measurements are collected in under a second, but typically several measurements per patch

and several patches per animal are collected, resulting in a temporal resolution spanning from several seconds to minutes. For multispectral photography, capture with limited spectral sensitivity (e.g. omitting ultraviolet light) can allow for video-speed capture, but increasing spectral sensitivity typically slows capture to the range of minutes per image (though see [7] for multispectral video including ultraviolet light). For hyperspectral imaging, a similar trade-off can occur: full-frame hyperspectral imagers with limited spatial and spectral sensitivity can capture images at video-speed. The hyperspectral imager used in the current paper has high spatial and spectral resolution, but each image takes several minutes to capture."

Figure 3. Would it please be possible to make the font all equal size? Axes in Fig 3A iii, C, and D are especially hard. On my end the "H" label in a iii shows up as "1I" instead. I know that this is a little challenging when making composite images; however, many of the axes are too small (even when considering that your final resolution will be higher). You should be able to choose a font size that will end up being the same size given the final figure size and the relative size of each panel.

We have updated figures 1, 3 – 7 to increase and homogenize font size where possible.

Line 373: suggested reword in brackets "… but this came at the cost of not having a uniform sampling density (samples per cm2, for instance) [, which] may be more important for other research questions."

Thank you. We've made the suggested edit.

Line 485: I would recommend removing "spatially" before 2d in this sentence.

We've followed this advice. The reason it was there was because we were avoiding confusion with calling a data-cube just '2D', but I think we probably only increased confusion.

Line 507: Please be cautious. See my comment on Fig. 1. Fig 3 seems to have reasonable RGB values, likely generated from image-generated spectra (however, is D devoid of brightness information, while C has brightness and color information?). In any case, be cautious here as there appeared to be an issue with one RGB image.

We generate RGB colors from spectra throughout; see response to comment above. Fig 3D is displaying the same spectra as Fig 3C, but colored only according to the legend to the left of Fig 3B (Green: King bird-of-paradise, Blue: hybrid King of Holland's bird-of-paradise, Orange: Magnificent bird-of-paradise), hence no brightness or color information.

Fig 4. Like in Fig. 1 this appears saturated, and I'm 99% sure this isn't correct. That is, none of your raw data are saturated and you should be able to produce a fine image with a very high degree of color fidelity.

Figure 4 A is generated completely separately to the hyperspectral images – the RGB colors here come from a mosaic of the values from the (unmodified consumer camera) images used to generate the 3D models. I suspect that part of this problem is partly a display issue – potentially due to the rendering of the image in the .pdf generated for review. I've slightly modified the levels of this diagrammatic image to lower saturation.

Line 544: Of [each] specimen? Didn't you do this with more than one specimen, even if much of the focus is on one specimen? Also, I'm not sure that "Thus, hyperspectral imaging is a single tool that simultaneously produces data with high spectral and high spatial resolution" follows directly from your prior sentence. Perhaps instead, "Thus, [we illustrate the utility of] hyperspectral imaging [as] a [] tool [to] simultaneously [produce] data with high spectral and high spatial resolution" Or similar

We have made these changes.

Fig. 5. Lovely image! Are the spectral peaks shown in K expected? I cannot really read to y-axes, but assume these seem reasonable to you in comparison with spectroscopy of a similar measurement geometry.

As above, we've tried to deal with small axes throughout. We believe the low values of peak reflectance here <10% for the green breast plumage is due to iridescence; at the angle we photographed only some pixels will be brightly reflecting light from the source to the camera (as noted in the section below starting "interpreting the magnitude of the peak reflectance is more difficult…"). The spectral peaks are expected in the sense that this is a structurally colored green patch.

Line 591: Given the striking peak height for the hybrid, I think that it would be important to highlight that you are talking about the λ_{max} value for these curves. This should avoid unnecessary confusion. A vertical line at the λ_{max} for the hybrid could also help the reader see that the hybrid falls intermediate to the two parent species.

We have made this edit to the text, and we think that this has helped a great deal with clarity here.

Lines 671: With respect to statements such as "more saturated and slightly redder in hue than the hybrid" here and throughout, should be supported by test statistics. Even though you have only a handful of specimens, you have a large population of data points from both specimens (per patch) and you have all the metrics needed (e.g., distance from the achromatic point may be a metric of saturation). This goes for other similar statements (e.g., 679-700, etc.). Unless I'm missing something, it seems reasonable to determine whether certain color metrics are, or are not, greater.

We appreciate this comment. However, we have explicitly (L395) taken a descriptive over a statistical approach, and (in line with reviewer and editor comments), we have now reduced the focus on the specific example of the birds-of-paradise in favor of concentrating on the pipeline. We feel that adding adding summary statistics (or test statistics) will shift the focus back toward quantitative results of the of the Bird-of-Paradise case study, which is probably not desirable. Therefore, we prefer to retain our more qualitative interpretation of the data in the figure here and elsewhere.

Line 886: It would be important to note that this figure of $55,000 is the base fee, and a full setup may be out reach for many researchers for many years.

This line has been updated to:

"Hyperspectral imaging systems remain expensive. Resonon is currently selling its UV-sensitive imaging system for about $55,000 USD, and the light source and lens used here increase the total cost to around $75,000 USD."

Line 897: I found this switch back to the bird-of-paradise a little abrupt. I'm not 100% sure how to best address that, but perhaps some subtle shuffling of sections or connective phrases could help. That said, overall, the tone of the manuscript is striking a much better balance between the new method and the biological significance (in my opinion).

We feel that having this section start under the subtitle "Insights into the plumage color of a hybrid Bird-of-Paradise" should help the reader make sense of the move back to the discussion of our bird-of-paradise example. We're glad you found the MS improved. Thank you for pointing us in that direction.

Lines 946-960: I would be a little cautious here because even if a light source matches natural lighting, spectroscopic measurements (and those from imaging) would capture radiance, rather than reflectance per se (inferred or otherwise). You could get reflectance by using a monochromator, thus separating out the reflectance from the emission spectra, as is typical in the large Perkin spectrometers used in many chemistry classrooms. Importantly, many (but not all) museum specimens were treated with chemicals like arsenic and borax, which are known to fluoresce. One potential simple solution would be to suggest replicating your work on fresh, untreated, specimens to confirm and characterize the fluorescence (for an example, please see Travouillon et al. 2023 https://doi.org/10.1[1]098/rsos.230325).

Thank you for your thoughtful comment. We have omitted the caveat about light sources, opting for a more accurate phrasing, and we added a reference to the paper you suggested. What we mean here is only that inferred reflectance (generated from radiance) can be influenced by fluorescence, and we later note that this could be investigated using a monochromator as you point out. This section now reads:

"Neither the King nor the hybrid bird-of-paradise shows significant fluorescence: whatever the mechanism, we do not see evidence of intermediacy in the hybrid. We note that strong fluorescence can complicate the measurement of animal color; the light produced by fluorescence can introduce errors in inferred reflectance (pers. comm. Jolyon Troscianko). Exactly what mechanism generates fluorescence in the Magnificent bird-of-paradise, and why it appears absent in the hybrid, could be investigated by sectioning and imaging feather samples—and by testing for any influence of specimen preparation methods [97]. Further characterization of the fluorescence, including specifics of the excitation and emission wavelengths, might also be studied using a hyperspectral imager in concert with spectral filters and/or a monochromator."

Reviewer #3 (Jolyon Troscianko, signs review): Overall the authors have done a very good job at addressing my concerns, and those of the other reviewers, making a number of significant changes to the manuscript and analyses. Although the authors haven't provided new evidence that this method is superior to others (e.g. benchmarking), their discussion provides readers with enough information to make an informed judgement, and the method is a valuable addition to the field.

Dr. Troscianko: Thank you for your detailed initial review and for taking another look this time around. We're glad you feel that we're better informing a reader about the various methods open to them.

Minor points:

Re. the addition of temporal resolution to table 1, I would say that spectrometers should be high (they measure all the dimensions they can measure near-instantaneously). Multispectral should be high for RGB, and medium for wider spectral bands, and hyperspectral should be medium or low. Obviously, hyperspectral methods must trade off the dimensions against each-other, so will typically be slower. Otherwise, you could specify that the table only refers to techniques in the vis-UV range.

Thank you for this comment. Given this, and Dr. Hanley's comment on the same row of the table, we feel that omitting this row is probably best. See response above, but in short – as you note here – no one simple entry is going to be fair to any technique - covering all use-cases and

hardware. We add some note of the trade-offs and variability in temporal resolution/time to sample an animal in the table caption where there's more space for nuance:

"In terms of temporal resolution, we note that the amount of time to sample an individual animal is highly variable both within and among techniques. For spectrophotometry, individual measurements are collected in under a second, but typically several measurements per patch and several patches per animal are collected, resulting in a temporal resolution spanning from several seconds to minutes. For multispectral photography, capture with limited spectral sensitivity (e.g. omitting ultraviolet light) can allow for video-speed capture, but increasing spectral sensitivity typically slows capture to the range of minutes per image (though see [7] for multispectral video including ultraviolet light). For hyperspectral imaging, a similar trade-off can occur: full-frame hyperspectral imagers with limited spatial and spectral sensitivity can capture images at video-speed. The hyperspectral imager used in the current paper has high spatial and spectral resolution, but each image takes several minutes to capture."

Regarding the spectral filtering, FigS2 is useful, and the raw spectra don't look bad. The opsin curves applied to these raw spectra will be smooth, and will also have the effect of smoothing out the noise substantially. In any case, the effects are now easy for the reader to see.

We are glad that you feel the addition was worthwhile!

Regarding costs, thanks for adding these - pretty pricey! I would disagree that costs are coming down though. Off-the-shelf hyperspectral imagers simply don't have a large enough consumer base, and UV optics and light sources haven't become cheaper in over the past 15 years or so I've been paying attention. Given the renewed focus on methods, I would still recommend you add an estimate of the full cost, presumably nearer $70k?

We have modified this sentence in line as follows:

"Hyperspectral imaging systems remain expensive. Resonon is currently selling its UV-sensitive imaging system for about $55,000 USD, and the light source and lens used here increase the total cost to around $75,000 USD."
